# Immunological Features and Potential Biomarkers of Systemic Sclerosis–Associated Interstitial Lung Disease and Idiopathic Pulmonary Fibrosis

**DOI:** 10.1111/crj.70072

**Published:** 2025-03-31

**Authors:** Shuai Shao, Siyu Cao, Yusha Chen, Zhijin Zhang, Tong Zhaohui

**Affiliations:** ^1^ Department of Respiratory and Critical Care Medicine, Beijing Institute of Respiratory Medicine, Beijing Chao‐Yang Hospital Capital Medical University Beijing China

**Keywords:** CD4/CD8 ratios, disease progression, IPF, SPA17, SSc‐ILD, T cells

## Abstract

**Background:**

This study aims to summarize the similarities and differences in immune cell characteristics, and potential therapeutic targets between systemic sclerosis‐associated interstitial lung disease (SSc‐ILD) and idiopathic pulmonary fibrosis (IPF).

**Methods:**

This study included SSc‐ILD and SSc‐nonILD patients who were admitted to Beijing Chaoyang Hospital between April 4th, 2013, to June 30th, 2023. Publicly available datasets, including peripheral blood monocular cell (pbmc) single‐cell data, SSc, SSc‐ILD pbmc transcriptome data, and SSc‐ILD, IPF lung tissue transcriptome data were analyzed. Statistical analyses were conducted using the SPSS and R software, employing standard statistical methods and bioinformatics packages such as Seurat, DESeq2, enrichR, and CellChat.

**Results:**

The results revealed that the CD4+/CD8+ T cell ratio of pbmc in SSc‐ILD patients was significantly higher than in SSc‐nonILD patients. In IPF patients, an elevated CD4+/CD8+ T cell ratio was also observed in progressive group, and Treg and mature CD4+ T cells might cause this change. JAK–STAT pathway and the cytokine–cytokine receptor interaction pathway were activated in peripheral blood T cells of IPF patients. The CD30, CD40, and FLT3 signaling pathways were found to play crucial roles in T cell interactions with other immune cells among IPF patients. SPA17 as a commonly upregulated gene among SSc, SSc‐ILD, and IPF pbmc and lung, with its expression correlating positively with disease severity and lung function progression.

**Conclusion:**

CD4+/CD8+ T cell ratio might associate with ILD initiation and progression; Treg cells and mature CD4+ T cells play key roles of it. SPA17 might serve as a pan‐ILD marker and associated with lung function progression.

## Graphical abstract

- The results revealed that the CD4+/CD8+ T cell ratio of pbmc in SSc‐ILD patients was significantly higher than in SSc‐nonILD patients. In IPF patients, an elevated CD4+/CD8+ T cell ratio was also observed in progressive group, and Treg and mature CD4 + T cells might cause this change.
- The CD30, CD40, and FLT3 signaling pathways were found to play crucial roles in T cell interactions with other immune cells among IPF patients.
- SPA17 as a commonly upregulated gene among SSc, SSc‐ILD, and IPF pbmc and lung, with its expression correlating positively with disease severity and lung function progression.

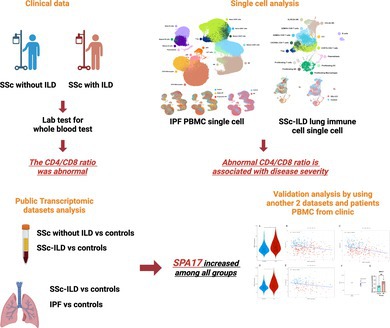

**Abbreviations**
ACR: American College of Rheumatology
ALT: alanine aminotransferase
AST: aspartate aminotransferase
AUC: area under the curve
BAL: bronchoalveolar lavage fluid
COVID‐19: novel coronavirus disease 2019
CLR: CRP‐to‐lymphocyte ratio
CRP: C‐reactive protein
DEG: differentially expressed gene
DLCO: diffusing capacity for carbon monoxide
ECM: extracellular matrix
EULAR: European League Against Rheumatism
FT3: free triiodothyronine
FT4: free thyroxine
FVC: forced vital capacity
GEO: Gene Expression Omnibus
HRCT: high‐resolution computed tomography
ILD: interstitial lung disease
IPF: idiopathic pulmonary fibrosis
IQR: interquartile range
JAK–STAT: Janus Kinase‐Signal Transducer and Activator of Transcription
KEGG: Kyoto Encyclopedia of Genes and Genomes
LMR: lymphocyte‐to‐monocyte ratio
miR: microRNA
NLR: neutrophil‐to‐lymphocyte ratio
PBMC: peripheral blood mononuclear cell
qPCR: quantitative polymerase chain reaction
ROC: receiver operating characteristic
SSc: systemic sclerosis
SSc‐ILD: systemic sclerosis–associated interstitial lung disease
TNFRSF: tumor necrosis factor receptor superfamily
TNFSF: tumor necrosis factor superfamily
TSH: thyroid‐stimulating hormone

## 1 Background

Systemic sclerosis (SSc) is an autoimmune disorder characterized by various clinical manifestations, including fibrosis of the skin and internal organs [1]. Interstitial lung disease (ILD) remains a primary cause of mortality among SSc patients [2], affecting 35%–52% of cases and contributing to 20%–40% of deaths [3, 4]. Idiopathic pulmonary fibrosis (IPF) is another severe form of ILD characterized by rapid disease progression and worsening pulmonary scarring, with a median survival of only 2–3 years [5, 6]. Although both SSc‐ILD and IPF manifest as ILD, they differ significantly in pathological features, underlying mechanisms, and treatment responses. Despite active pharmacological treatment for both SSc‐ILD and IPF, a considerable proportion of patients continue to experience disease progression and mortality [7, 8, 9, 10]. The significant heterogeneity and variability of SSc‐ILD among individuals have led to an incomplete understanding of the disease, resulting in the adaptation of therapeutic strategies from IPF treatment for managing SSc‐ILD. Nevertheless, the available treatment options for these diseases remain limited and require further advancement.

Peripheral blood immune cells are reported strongly associated with disease activity and progression [11, 12]. The CD4+/CD8+ T cell ratio is frequently employed as a marker for evaluating the balance of the immune system [11, 12]. Among CD4+ T cells, the expression of chemokine receptors CXCR3 and CCR4 is commonly used to distinguish Th1 and Th2 cells, respectively, and an imbalance in Th1/Th2 responses is believed to play a key role in the pathogenesis of IPF [13, 14]. In ILD, CD8+ T cells are known to diffusely infiltrate the parenchyma of fibrotic IPF tissues and can differentiate into subtypes that either produce IFN‐γ but not IL‐4 (which may mitigate fibrosis) or IL‐4 but not IFN‐γ (which could promote fibrosis) [15]. As a result, CD8+ T cells may exert opposing effects on fibrosis, with complex and precise regulatory mechanisms maintaining this balance. Previous studies also suggest that aging‐associated dysfunction in T cell development and maturation may contribute to an increased CD4+/CD8+ T cell ratio [16, 17]. Research involving patients with MDA5‐ILD has shown that an elevated CD4+/CD8+ T cell ratio is correlated with disease onset and progression [18]. However, the relevance of the CD4+/CD8+ T cell ratio in other forms of ILD remains unclear. Moreover, although peripheral blood T cell dysfunction is frequently implicated in ILD pathogenesis, whether T cell function varies with ILD severity is still unknown.

This study utilizes a comprehensive approach that integrates 10‐year clinical data, single‐cell sequencing, and transcriptomic analysis to address the complexities and potential roles of CD4+/CD8+ T cell ratio and disease‐associated functions in the context of SSc‐ILD and IPF. Through these methods, we aim to deepen the understanding of immune cell involvement in these two diseases, providing new insights into the underlying mechanisms and potential biomarkers of ILD.

## 2 Method

### 2.1 Single‐Cell Sequence

#### 2.1.1 Analysis Database

To investigate the peripheral blood profile of IPF patients compared with matched healthy controls, we downloaded the publicly available dataset GSE233844 from the NCBI Gene Expression Omnibus (GEO) database [5]. Due to the de‐identified nature of this publicly available data, it is exempt from institutional ethics review board oversight and does not require informed patient consent. The original research constructed a 10× library using a five‐primer approach and employed Illumina HiSeq 4000 ( *Homo sapiens* ) and Illumina NovaSeq X ( *Homo sapiens* ) for sequencing. After downloading the fastq files, we performed upstream data processing using the CellRanger aggr pipeline (v.7.1.0), referencing the GRCh38‐2020‐A genome. For SSc‐ILD patients, due to lack of qualified pbmc single cell data that we can assess, we downed SSc‐ILD data (GSE128169) to analyze the CD4+/CD8+ T cell ratio in lung tissue to provide more details among SSc‐ILD patients [1]. This dataset includes 16 samples from different lung locations of six SSc‐ILD patients and five control subjects from different lung locations.

#### 2.1.2 Data Analysis

For quality control, we removed low‐quality cells and doublets. Cells with an nCount_RNA greater than 2000 and a mitochondrial gene proportion below 15% from IPF pbmc dataset were included in the analysis. Cells with an nCount_RNA count exceeding 300 and mitochondrial gene content below 20% from SSc‐ILD lung dataset were included in the analysis. Data normalization was performed using Seurat v5.1.0. We applied the “vst” method and identified 2000 highly variable genes using the FindVariableFeatures function. Subsequently, we scaled the data and conducted principal component analysis (PCA) using RunPCA. Integration was performed by rPCA. Dimensionality of the PCs was determined using the Jackstraw plot, with careful examination to ensure selected PCs were not influenced by background noise. We then applied FindNeighbors (k.param = 20) and FindClusters (resolution = 1.2 for IPF pbmc dataset and 1.5 for SSc‐ILD lung dataset) for clustering. UMAP plot was used to visualize the clusters in two dimensions. Differentially expressed genes (DEGs) for each cluster were identified using the FindAllMarkers function in Seurat. Manual curation and the Azimuth tool were used for cell type annotation.

Differential composition and gene expression analyses were performed to identify disease‐specific and shared changes. To ensure the reproducibility of identified DEGs across individuals within the same group, we used the AggregateExpression function to pseudobulk counts by donor‐condition‐cell type, producing sample‐level DEGs for downstream analysis. The comparison of cell proportions and statistical analyses were conducted using the Wilcoxon test. Pathway enrichment analysis was carried out using the enrichR package and checked with Gene Ontology website (https://geneontology.org/). Cell–cell communication analysis was conducted using the CellChat R package [19].

### 2.2 Transcriptomic Database and Analysis Methods

The inclusive and exclusive criteria for us to choose datasets are the following: Inclusion Criteria: The dataset must be publicly available and include clearly annotated samples of SSc, SSc‐associated interstitial lung disease (SSc‐ILD), IPF, or healthy controls. The data must contain high‐quality bulk RNA sequencing information suitable for downstream analysis. The collection methods and experimental procedures must adhere to established standards (e.g., using 10× Genomics platforms or Illumina high‐throughput sequencing platforms). Exclusion Criteria: These include datasets lacking clear pathological group annotations (e.g., no distinction between SSc‐ILD and SSc‐nonILD), sample sizes that are too small (fewer than 50 patient samples) or datasets without healthy controls, datasets with poor quality (e.g., high proportions of low‐quality cells or missing sequence data), and inclusion of samples from patients with undefined diagnoses or comorbidities that could contribute to pulmonary conditions (e.g., malignancies, infectious diseases). Currently, there is no publicly available single‐cell peripheral blood mononuclear cell (PBMC) database for SSc‐ILD. Therefore, we downloaded peripheral blood transcriptomic data from SSc‐only (GSE117928) [20] and SSc‐ILD (GSE231691) [21] patients and their matched healthy controls. Additionally, we retrieved lung transcriptomic data for IPF, SSc‐ILD, and non‐ILD controls (GSE231693) [21]. RNA‐seq reads for each sample were aligned using GRCm38 with STAR (v2.7.3a). The DESeq2 package was used to identify differentially expressed genes (DEGs). Pathway analysis and GO term enrichment analysis were conducted using Enrichr. Genes were considered upregulated if log2 FoldChange ≥ 0.5 and *p*‐adjusted value < 0.05, and downregulated if log2 FoldChange ≤ −0.5 and *p*‐adjusted value < 0.05. Pathway enrichment analysis was performed using the clusterProfiler package [v4.4.4].

### 2.3 Clinical Data

#### 2.3.1 Data Sources and Population

This study utilized an automated extraction of patient records from Beijing Chaoyang Hospital's electronic medical record system, covering SSc‐nonILD and SSc‐ILD cases from April 4th, 2013, to June 30th, 2023. Due to the limitations of the retrospective system, we could not access information on patients from health examination centers; hence, peripheral blood comparisons between healthy individuals and IPF patients were not conducted. Following automated data extraction, two authors independently verified the data. Additionally, an experienced respiratory clinician manually reviewed the quality of included patients and their adherence to the inclusion criteria to ensure data authenticity and reliability.

The inclusion criteria were (1) diagnosis of SSc based on the 2013 updated classification criteria by the European League Against Rheumatism (EULAR) and the American College of Rheumatology (ACR) [22], incorporating clinical, radiological, and serological findings; (2) diagnosis of SSc‐ILD based on respiratory symptoms and interstitial changes observed on high‐resolution computed tomography (HRCT); (3) patients aged over 18 years; (4) first‐time hospital admission; and (5) no prior use of corticosteroids, immunosuppressants, or antifibrotic treatments. Exclusion criteria included (1) pregnancy, (2) known or suspected infections, (3) active malignancies, (4) undifferentiated connective tissue disease, (5) overlap syndrome, (6) inability to obtain blood routine results within 48 h of admission, and (7) transfer to the ICU within 24 h of admission. This study was approved by the Ethics Committee of Beijing Chaoyang Hospital (2023‐ke‐355). Informed consent was waived as all data were de‐identified and extracted from an anonymous system.

#### 2.3.2 Data Collection and Quality Control

The data extracted from the electronic medical record system included patient age, sex, comorbidities, smoking history, laboratory tests (e.g., complete blood count, biochemical tests), and pulmonary function results. Additionally, the study retrieved the CT reports of each patient's lungs, which were further reviewed by a respiratory specialist to confirm the accuracy of the ILD or non‐ILD diagnosis.

#### 2.3.3 Sample Collection

PBMCs used in this study were obtained from patients diagnosed with IPF in the outpatient clinic. After explaining the study's purpose and the potential benefits it may bring to clinical practice, 5 mL of peripheral blood was collected from each participant following the signing of informed consent. PBMCs were isolated using lymphocyte separation medium (Beyotime, C0025–200 mL). The separated plasma and PBMCs were de‐identified, coded, and stored at −80°C. This study protocol was approved by the Ethics Committee of Beijing Chao‐Yang Hospital (2023‐ke‐355). Informed consent was obtained from all participants.

#### 2.3.4 Statistical Analysis

Statistical analyses were conducted using the SPSS 26.0 (IBM Corp., Armonk, NY) and R software (version 3.6.0). The Shapiro–Wilk test was applied to assess normal distribution; the results of Shapiro–Wilk test were provided in Supplementary Files 1. Continuous variables following a normal distribution were reported as mean ± standard deviation and compared between groups using the independent sample *t*‐test. For continuous variables not following a normal distribution, data were expressed as median and interquartile range (IQR) and analyzed using the Mann–Whitney *U* test. In the multivariable analysis, age, sex, smoking, diabetes, and hypertension were included as confounders to adjust for the CD4+/CD8+ T cell ratio. Categorical variables were presented as frequencies and percentages, with group differences evaluated using the chi‐square test or Fisher's exact test for smaller sample sizes. The study utilized receiver operating characteristic (ROC) curves and the area under the curve (AUC) to evaluate the predictive performance of variables in determining ILD. Cutoff values were calculated using Youden's index, considering sensitivity and specificity. A *p*‐value of less than 0.05 was considered statistically significant in all analyses.

### 2.4 qPCR

PBMC were separated from human peripheral blood by LSM™ Lymphocyte Separation Medium (MP Biomedicals, 0850494‐CF). Total RNA was extracted from pbmc using the TRIzol (Sigma) method, and 1000 ng total RNA was reverse transcribed using a PrimeScript RT Reagent Kit with gDNA Eraser (TAKARA, RR047A). qPCR was performed with Hieff UNICON Universal Blue qPCR SYBR Green Master Mix (YEASEN, 11184ES08) and a Real‐time PCR ABI7500. The primer sequences were as follows: Forward primer: CTGACACGCGAGATTCTGAGA. Reverse primer: CTGCTGGATCAAAGTTGGTTTTC. The results were normalized to GAPDH expression levels, and data were shown as the relative gene expression.

## 3 Results

### 3.1 Clinical Data Indicate an Association Between CD4/CD8 T Cell Ratio and SSc‐ILD Onset

From April 4, 2013, to June 30, 2023, 203 patients diagnosed with SSc were treated at Beijing Chaoyang Hospital. Of these, 22 patients were excluded due to repeated admissions, 49 had received treatment prior to admission, 10 lacked blood test results within 48 h of admission, and two were transferred to the ICU within 24 h. Ultimately, 93 SSc‐ILD and 27 SSc‐nonILD patients were included in the analysis. There were no statistically significant differences between the groups regarding age, sex, or comorbidities (*p* > 0.05), but significant differences were observed in peripheral blood cell composition. SSc‐ILD patients had higher C3 levels (90.60 [77.60–99.80] vs 72.40 [67.85–83.08], *p* = 0.04552) and C4 levels (18.60 [15.80–23.20] vs 16.20 [12.95–21.40], *p* < 0.001) compared with SSc‐nonILD patients. Neutrophil counts (3.65 [2.95–5.46] vs 2.62 [1.57–3.49], *p* = 0.00030), lymphocyte counts (1.76 [1.22–2.20] vs 1.45 [1.28–1.54], *p* = 0.02518), and neutrophil–lymphocyte ratio (NLR) (2.20 [1.65–3.24] vs 1.89 [1.67–2.26], *p* = 0.04490) were also higher in SSc‐ILD patients. The CD4+/CD8+ T cell ratio was notably higher in SSc‐ILD patients compared with SSc‐nonILD patients (1.75 [1.65–2.00] vs 1.11 [0.95–1.54], *p* < 0.001) (Table 1). Subsequently, we considered age, sex, smoking status, diabetes, and hypertension as confounders and performed a multivariable analysis for the CD4+/CD8+ T cell ratio. The results demonstrated that the CD4+/CD8+ T cell ratio remained statistically significant (OR = 6.79; 95% CI, 4.09–9.49; Padj < 0.001). More details can be found in Supplementary Files 1.

**TABLE 1:** Baseline characteristics of patients and clinical tests results.

	SSc‐ILD (*n* = 93)	SSc‐nonILD (*n* = 27)	*P‐value*
Demographic characteristics			
Age	58.01 ± 11.20	53.67 ± 14.26	0.154
Male (*n*, %)	15 (16.13%)	6 (22.22%)	0.565
Smoking status (*n*, %)	21 (22.58%)	3 (11.11%)	0.190
Drinking status (*n*, %)	9 (9.68%)	3 (11.11%)	0.731
Immunosuppressant (*n*, %)	24 (25.81%)	9 (33.33%)	0.441
Glucocorticoids (*n*, %)	42 (54.16%)	9 (33.33%)	0.274
Diabetes (*n*, %)	4 (4.30%)	3 (11.11%)	0.188
Hyperlipidemia (*n*, %)	3 (3.23%)	0 (0.00%)	0.999
Hypertension (*n*, %)	21 (22.58%)	3 (11.11%)	0.190
Lung function test			
DLCOcSB	4.40 (3.40–5.40)	5.01 (4.79–6.79)	**<0.001**
DLCOcSB (% pred)	43.00 (53.00–64.00)	75.00 (86.00–93.50)	**<0.001**
FEV1	1.86 (0.86–2.87)	2.02 (1.01–3.01)	**<0.001**
FEV1 (% pred)	72.00 (59.00–81.00)	90.00 (85.00–94.00)	**<0.001**
FVC	2.70 (1.65–3.75)	3.00 (2.10–3.82)	**<0.001**
FVC (% pred)	70.00 (60.00–80.00)	89.00 (83.50–95.00)	**<0.001**
Blood biochemistry tests			
ALT	19.00 (13.00–30.00)	26.00 (18.00–30.00)	0.148
Prealbumin	0.20 (0.16–0.26)	0.21 (0.09–0.23)	0.150
AST/ALT	1.30 (1.00–1.60)	1.40 (1.30–1.40)	0.683
AST	23.00 (18.00–30.00)	32.00 (25.00–37.00)	**0.005**
Albumin/globulin	1.20 (1.05–1.40)	0.95 (0.90–1.43)	0.219
TSH	2.08 (1.07–3.65)	2.34 (2.24–3.11)	0.663
FT3	2.78 (2.41–2.93)	2.80 (2.56–3.18)	0.392
FT4	1.13 (0.99–1.24)	1.03 (0.99–1.18)	0.379
C3	90.60 (77.60–99.80)	72.40 (67.85–83.08)	**<0.001**
C4	18.60 (15.80–23.20)	16.20 (12.95–21.40)	**0.046**
IgA	263.00 (213.00–367.00)	239.50 (182.50–424.25)	0.822
IgG	44214.32 (42174.33–44532.34)	43255.83 (41740.33–44771.33)	0.913
IgM	121.00 (89.70–177.00)	112.55 (85.03–147.00)	0.362
CRP	0.49 (0.28–0.89)	0.26 (0.19–0.77)	**0.026**
Leukocyte	6.10 (4.85–8.03)	4.28 (3.04–5.23)	**<0.001**
Neutrophil count	3.65 (2.95–5.46)	2.62 (1.57–3.49)	**<0.001**
Lymphocyte count	1.76 (1.22–2.20)	1.45 (1.28–1.54)	**0.025**
Monocyte count	0.40 (0.32–0.54)	0.45 (0.23–0.49)	0.475
Systematic inflammatory biomarkers			
CD3+CD4+/CD3+CD8+	1.75 (1.65–2.00)	1.11 (0.95–1.54)	**<0.001**
NLR	2.20 (1.65–3.24)	1.89 (1.67–2.26)	**0.045**
LMR	3.98 (2.87–5.64)	4.15 (2.79–5.40)	0.707
CLR	0.26 (0.13–0.65)	0.19 (0.12–0.45)	0.217
Clinical outcome			
LOS	10.00 (8.00–13.00)	8.00 (7.00–12.00)	0.081

*Note*: Bolded *p* values indicate statistical significance, meaning that *p* < 0.05.

Abbreviations: ALT, alanine aminotransferase; AST, aspartate aminotransferase; CLR, C‐reactive protein to lymphocyte ratio; CRP, C‐reactive protein; FT3, free triiodothyronine; FT4, free thyroxine; FEV1, forced expiratory volume in 1 s; FVC, forced vital capacity; LMR, lymphocyte to monocyte ratio; LOS, length of hospital stay; NLR, neutrophil to lymphocyte ratio; TSH, thyroid‐stimulating hormone.

**FIGURE 1:**
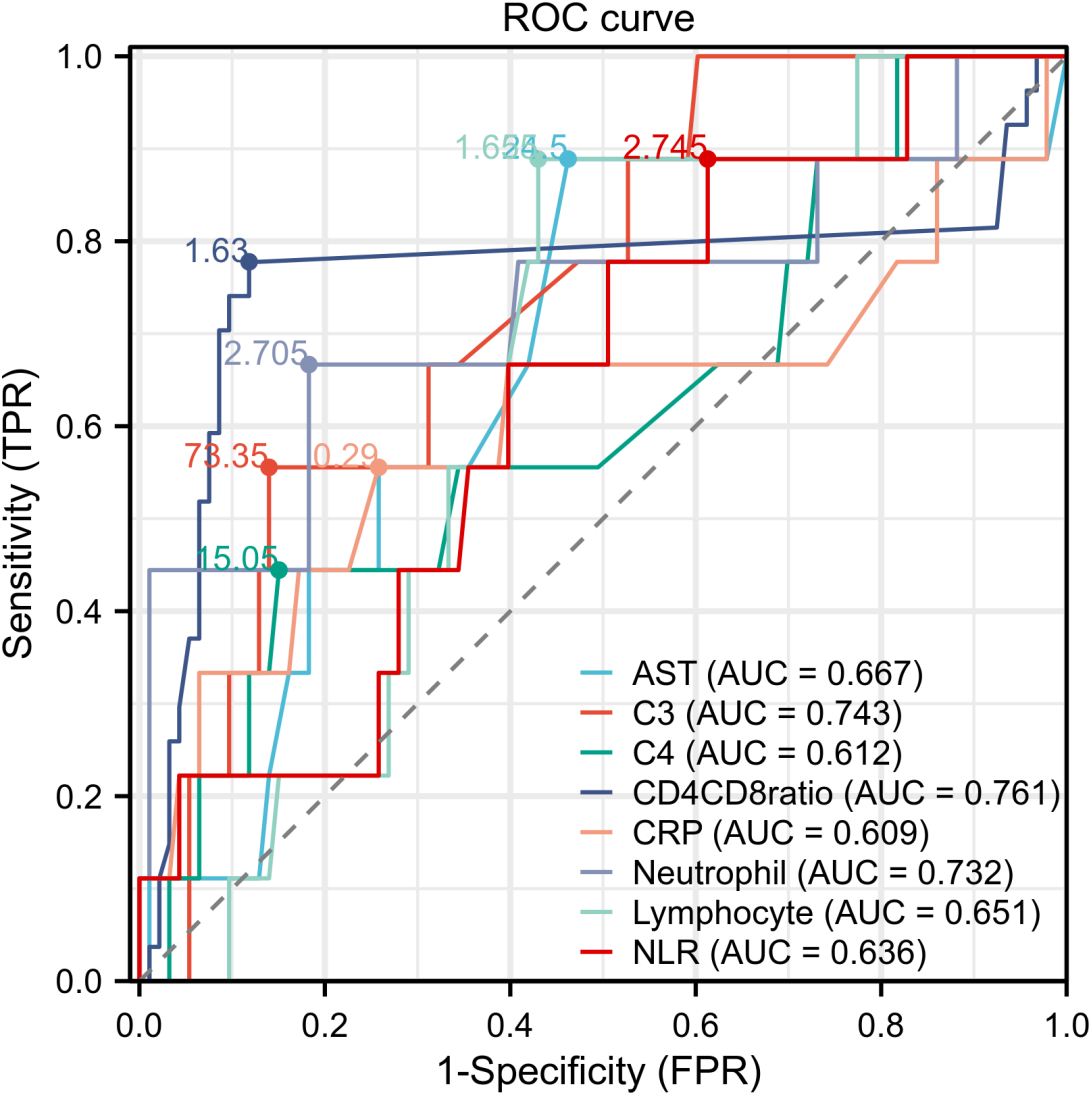
ROC curves of clinical data from SSc‐ILD patients. Operating characteristic (ROC) curves and the area under the curve (AUC) were used to evaluate the predictive performance of variables in determining ILD.

Further analysis of peripheral blood immune and inflammatory markers revealed that the CD4+/CD8+ T cell ratio demonstrated significant predictive power for distinguishing ILD presence (AUC = 0.761; Figure 1). These findings suggest that peripheral blood immune cell patterns in SSc‐ILD patients differ significantly from those in SSc‐nonILD patients, indicating that abnormal immune cell counts may be a critical reaction for the development and progression of SSc‐associated ILD disease.

### 3.2 Transcriptomic Data From Public Databases and Validation

#### 3.2.1 Single‐Cell Sequencing Data Indicate a Potential Association Between CD4/CD8 T Cell Ratio Among IPF and SSc‐ILD Patients

**FIGURE 2:**
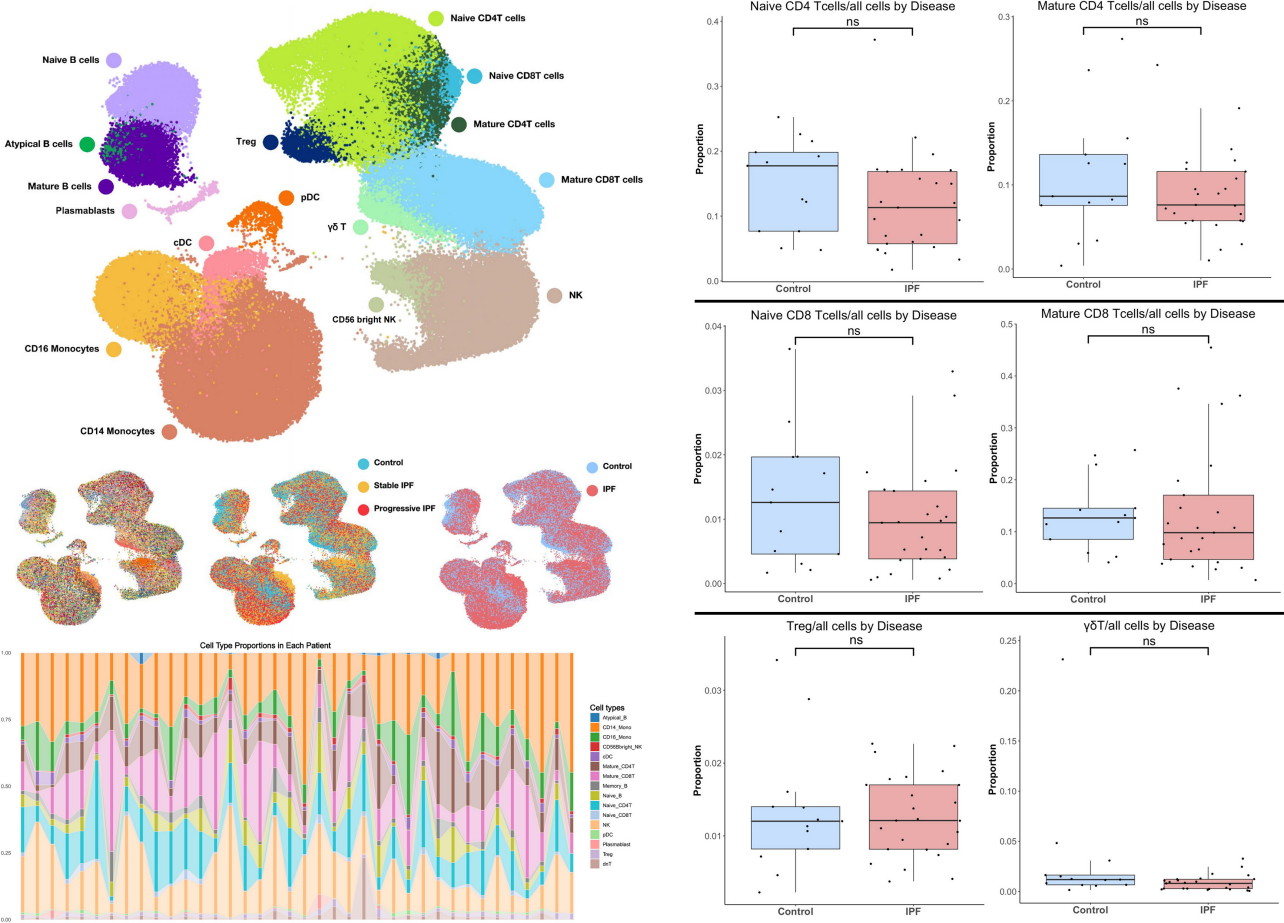
PBMC UMAP of IPF patients. (A)UMAPs of 183 101 pbmc cells from 12 progressive IPF patients, 13 matched stable IPF patients, and 13 matched controls without respiratory diseases labeled by cell type (top), subject (bottom left), disease status (bottom middle), and subject (bottom right). In the subject plot, each color depicts a distinct subject. (B) The bar plot of all cell types among 12 progressive IPF patients, 13 matched stable IPF patients, and 13 matched controls. (C) Box plots of different kinds of T cells between IPF and control groups. Cell proportions were calculated by Wilcoxon rank‐sum test; the results of Shapiro–Wilk test were provided in Supplementary Files 1. FDR adjustment was used to adjust *p* value. *p*‐value of less than 0.05 was considered statistically significant in all analyses. *p < 0.05, **p < 0.01, and ***p < 0.001.

**FIGURE 3:**
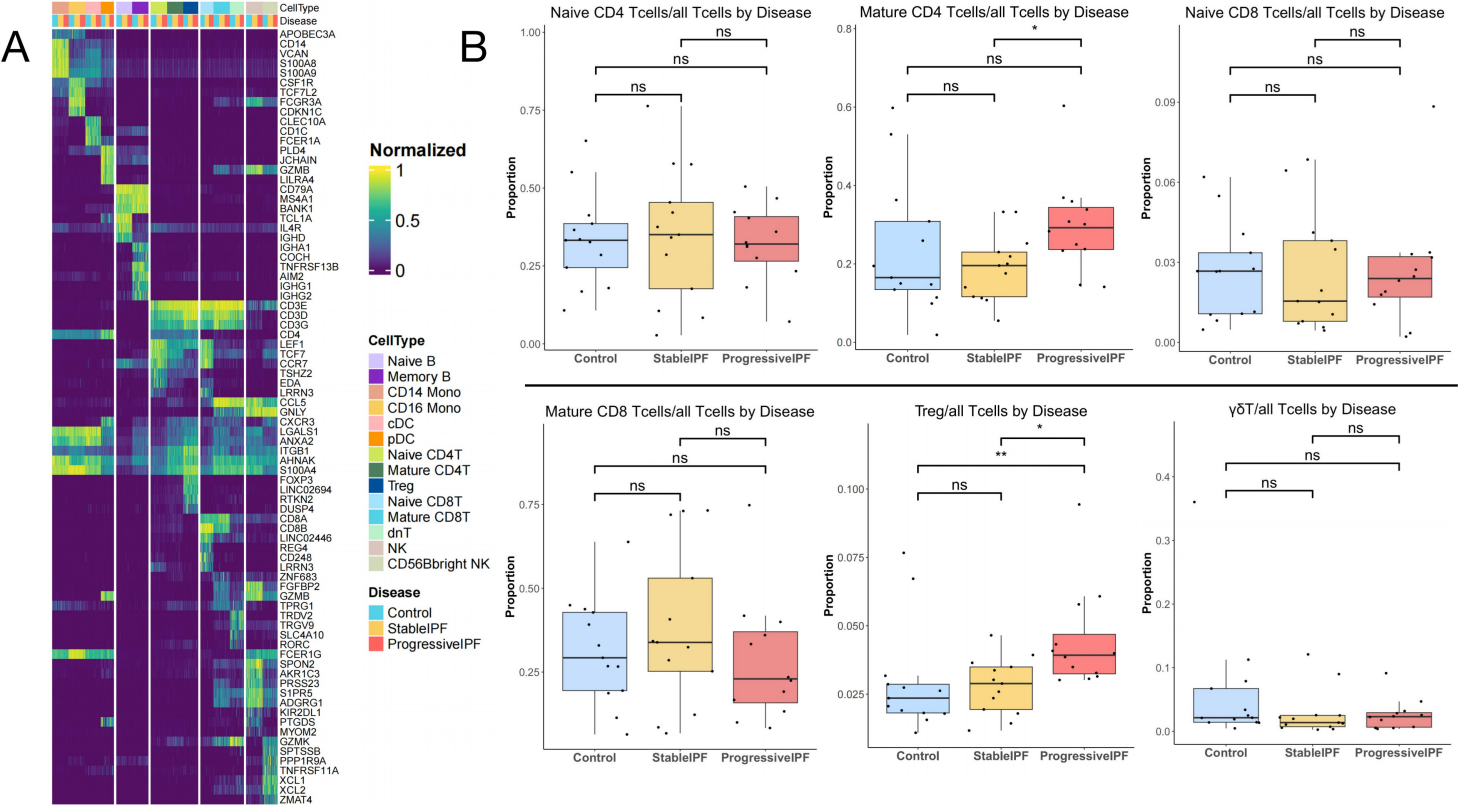
Heatmap of IPF pbmc cell types and box plots of different cell types. (A) Heat map representing characteristics of 14 subtypes of pbmc immune cells. Gene expression is unity normalized between 0 and 1 across pbmc cells. Each column represents an individual cell information regarding subject and disease state, and then cell type is represented in the colored annotation bars above. Each subject is represented by a unique color. (B) The box plots represent the proportion of a specific T cell subset relative to the total T cell population. Cell proportions were calculated by Wilcoxon rank‐sum test; the results of Shapiro–Wilk test were provided in Supplementary Files 1. FDR adjustment was used to adjust *p* value. *p*‐value of less than 0.05 was considered statistically significant in all analyses. *p < 0.05, **p < 0.01, and ***p < 0.001. dnT, double negative T cells.

The IPF single‐cell database included 12 progressive IPF patients, 13 matched stable IPF patients, and 13 matched controls without respiratory diseases. At the time of cohort inclusion, the IPF patients had not received any treatment. Progressive patients were defined as those who died within the 36‐month follow‐up period. After cell quality control and removal of doublets, the final Seurat object contained 183 101 cells across 30 clusters, encompassing 16 different cell types (Figure 2). Among T cell subgroup, there were statistically significant differences in Treg and mature CD4 T cells between the different groups (Figure 3).

**FIGURE 4:**
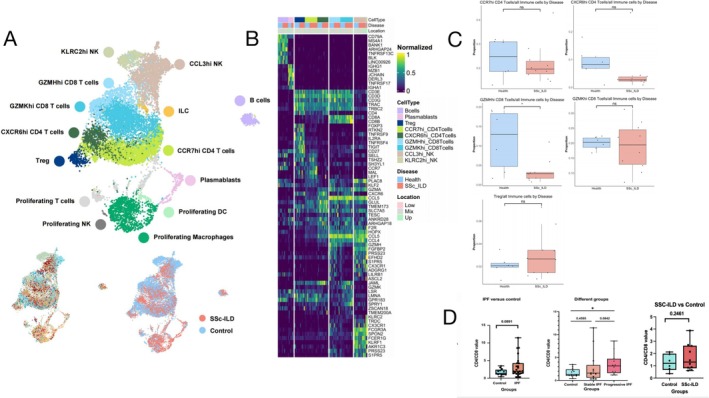
UMAP, heatmap, and boxplots of SSc‐ILD lungs versus controls, and CD4/CD8 T cell ratio among different diseases and conditions. (A) UMAPs of 11 520 lymphoid cells includes 16 samples from different lung locations of six SSc‐ILD patients and five control subjects labeled by cell type (top), subject (bottom left), and conditions (bottom right). (B) Heat map representing characteristics of 14 subtypes of SSc‐ILD immune cells. Gene expression is unity normalized between 0 and 1 across pbmc cells. Each column represents an individual cell information regarding subject and disease state, and then, cell type is represented in the colored annotation bars above. (C) The box plots represent the proportion of a specific T cell subset relative to the lymphoid cells population. Differences between groups were assessed using the Mann–Whitney *U* test. A *p*‐value of less than 0.05 was considered statistically significant in all analyses. (D) Left: CD4/CD8 T cell ratio among control and IPF patients. Middle: CD4/CD8 T cell ratio among control, stable IPF patients, and progressive IPF patients. Right: CD4/CD8 T cells ratio among control and SSc‐ILD patients. Cell proportions were calculated by Wilcoxon rank‐sum test; the results of Shapiro–Wilk test were provided in Supplementary Files 1. FDR adjustment was used to adjust *p* value. *p*‐value of less than 0.05 was considered statistically significant in all analyses. *p < 0.05, **p < 0.01, and ***p < 0.001.

We separated lymphoid cells that express PTPRC gene out from all the SSc‐ILD lung single‐cell dataset to help us focus on CD4/CD8 T cell ratios. After cell quality control and removal of doublets, the final Seurat object of SSc‐ILD included 11 520 cells 21 clusters, encompassing in 14 different cell types (Figure 4A,B). Although the number of different T cell types did not reach statistical significance between the SSc‐ILD and Control groups, we observed a clear trend of increased Tregs in the SSc‐ILD group, while GZMHhi CD8 T cells showed a marked decreasing trend in the SSc‐ILD group (Figure 4C).

To ensure comparability with clinical CD4/CD8 T cell ratios, we extracted the number of CD4‐expressing and CD8‐expressing T cells from the IPF pbmc single‐cell Seurat object and SSc‐ILD lung Seurat object to calculate the CD4/CD8 ratio (Supplementary File 1). The results showed no statistically significant difference in the CD4/CD8 T cell ratio between the stable group and the control group. Although the progressive group exhibited a trend of increased CD4/CD8 T cell ratio compared with the stable group, the difference was not statistically significant (*p* > 0.05). However, the CD4/CD8 T cell ratio in the progressive group was significantly higher than in the control group (3.1603 [1.8396–4.3890] vs. 1.5902 [0.8537–2.396], *p* = 0.0110; Figure 4D, left and middle figures). Although the difference in CD4/CD8 T cell ratio between SSc‐ILD and controls in the SSc‐ILD lung dataset did not reach statistical significance, this study found a clear trend of increased CD4/CD8 T cell ratio in SSc‐ILD lungs (1.3071 [0.8524–2.5038] vs 1.2038 [0.8209–1.8122], *p* = 0.2461) (Figure 4D right figure).

#### 3.2.2 Identification of Genes Related to IPF Progression in T Cells Using Single‐Cell Databases

**FIGURE 5:**
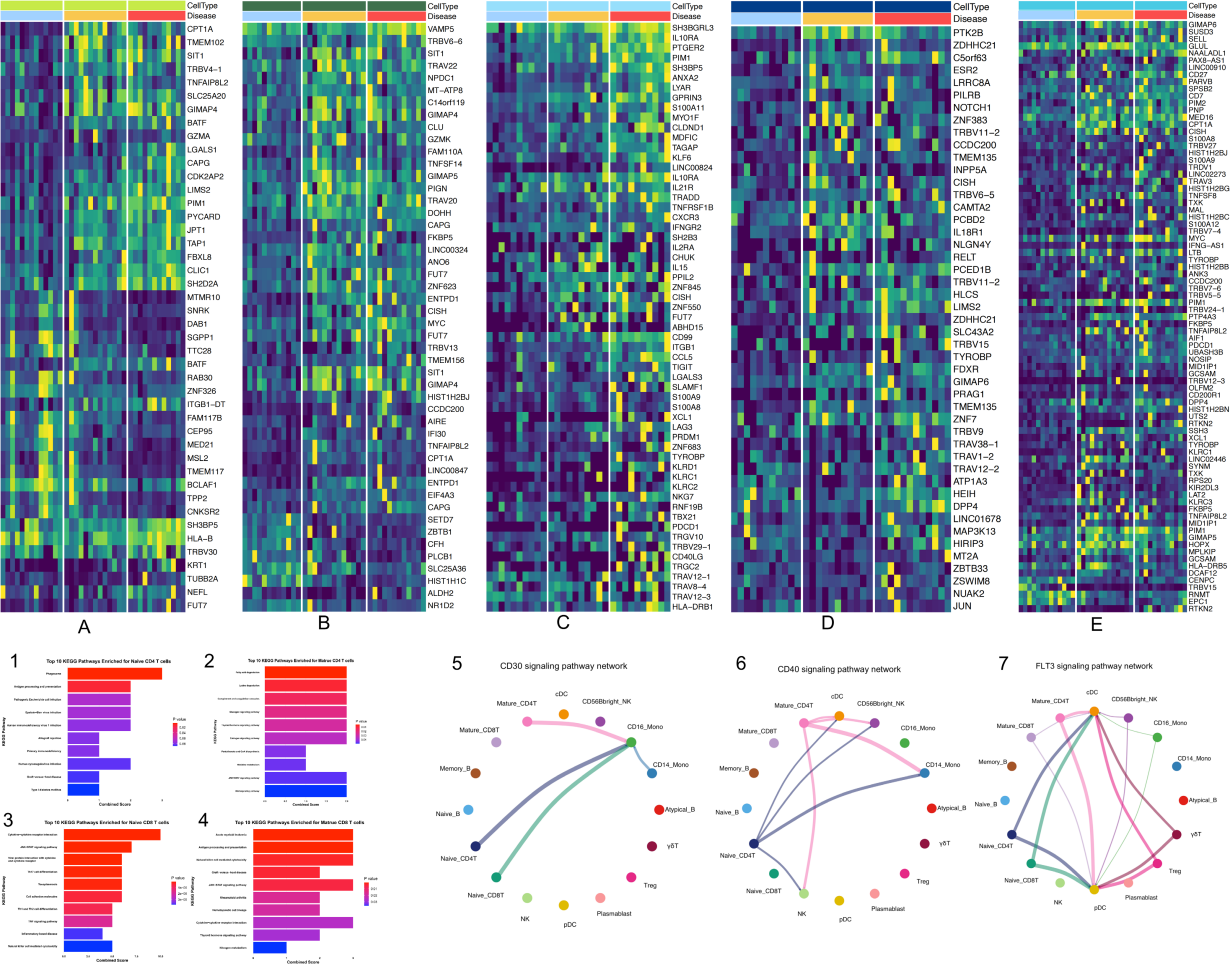
Deferential genes expression, pathway enrich analysis, and cell–cell talk analysis among IPF single cell dataset. (A) Heatmap shows the differential gene expression among control, stable IPF, and progressive IPF in naive CD4+ T cells; disease bar shows controls in blue, stable IPF in yellow, and progressive IPF in red. (B) Heatmap shows the differential gene expression among control, stable IPF, and progressive IPF in mature CD4+ T cells; disease bar shows controls in blue, stable IPF in yellow, and progressive IPF in red. (C) Heatmap shows the differential gene expression among control, stable IPF, and progressive IPF in naive CD8+ T cells; disease bar shows controls in blue, stable IPF in yellow, and progressive IPF in red. (D) Heatmap shows the differential gene expression among control, stable IPF, and progressive IPF in mature CD8+ T cells; disease bar shows controls in blue, stable IPF in yellow, and progressive IPF in red. (E) Heatmap shows the differential gene expression among control, stable IPF, and progressive IPF in Treg cells; disease bar shows controls in blue, stable IPF in yellow, and progressive IPF in red. (1) KEGG analysis of the differential gene expression among control, stable IPF, and progressive IPF in naive CD4+ T cells. (2) KEGG analysis of the differential gene expression among control, stable IPF, and progressive IPF in mature CD4+ T cells. (3) KEGG analysis of the differential gene expression among control, stable IPF, and progressive IPF in naive CD8+ T cells. (4) KEGG analysis of the differential gene expression among control, stable IPF, and progressive IPF in mature CD4+ T cells. (5)Cell‐talk hierarchy plot of CD30 signaling pathway network among IPF. (6) Cell‐talk hierarchy plot of CD40 signaling pathway network. (7) Cell‐talk hierarchy plot of FLT3 signaling pathway network.

To investigate differences beyond numerical changes, we analyzed the DEG in CD4+ and CD8+ T cells across the stable, progressive, and control groups at the sample level. We identified reproducible and disease‐associated gene patterns in various CD4+ and CD8+ T cell subsets (Figure 5). Notably, in CD8 naive T cells, we found a panel of genes associated with disease progression, including SH3BGRL3, IL10RA, PTGER2, PIM1, SH3BP5, and GPRIN3 (Figure 5C); there increased gradually from control to stable IPF to progressive IPF. Although Treg cells showed significantly increased trend among stable and progressive IPF patients, the gene expression does not change as obvious as CD4+ T cells and CD8+ T (Figure 5E), which suggested that the changes of counts of pbmc might isolate from the gene function.

Subsequent KEGG pathway enrichment analysis of the identified DEGs indicated significant activation of the JAK–STAT and cytokine–cytokine receptor interaction pathways in IPF associated T cells, particularly in naive CD8+ T cells (Figures 5‐3). Cell–cell communication analysis of pbmc of IPF patients highlighted that the CD30 pathway (TNFSF8 and TNFRSF8), CD40 pathway (CD40LG, ITGA5, ITGAM, ITGB1, ITGB2), and FLT3 signaling pathway (FLT3LG and FLT3) play crucial roles in the interactions between CD4+ and CD8+ T cells and other peripheral immune cells among IPF patients (Figure 5‐5/6/7, Supplementary Files 2, KEGG pathway result can be found in Supplementary Files 2).

#### 3.2.3 Transcriptomic Data From Public Platforms Reveals Disease‐Enriched Pathways in Peripheral Blood of SSc‐ILD Patients

Due to the lack of publicly available and reliable single‐cell sequencing data for peripheral blood of SSc‐ILD patients, we explored transcriptomic differences at the gene level between SSc‐ILD and SSc‐nonILD patients using public transcriptomic databases. We acquired data from GSE117928, which includes pbmc samples from 18 SSc patients and 19 matched healthy controls, as well as GSE231691, which comprises pbmc samples from 49 SSc‐ILD patients and 18 matched healthy controls. To further compare transcriptomic differences between SSc‐ILD and IPF patients' lungs, we included data from GSE231693, which consists of 20 SSc‐ILD patients, 20 IPF patients, and 20 matched healthy controls.

**FIGURE 6:**
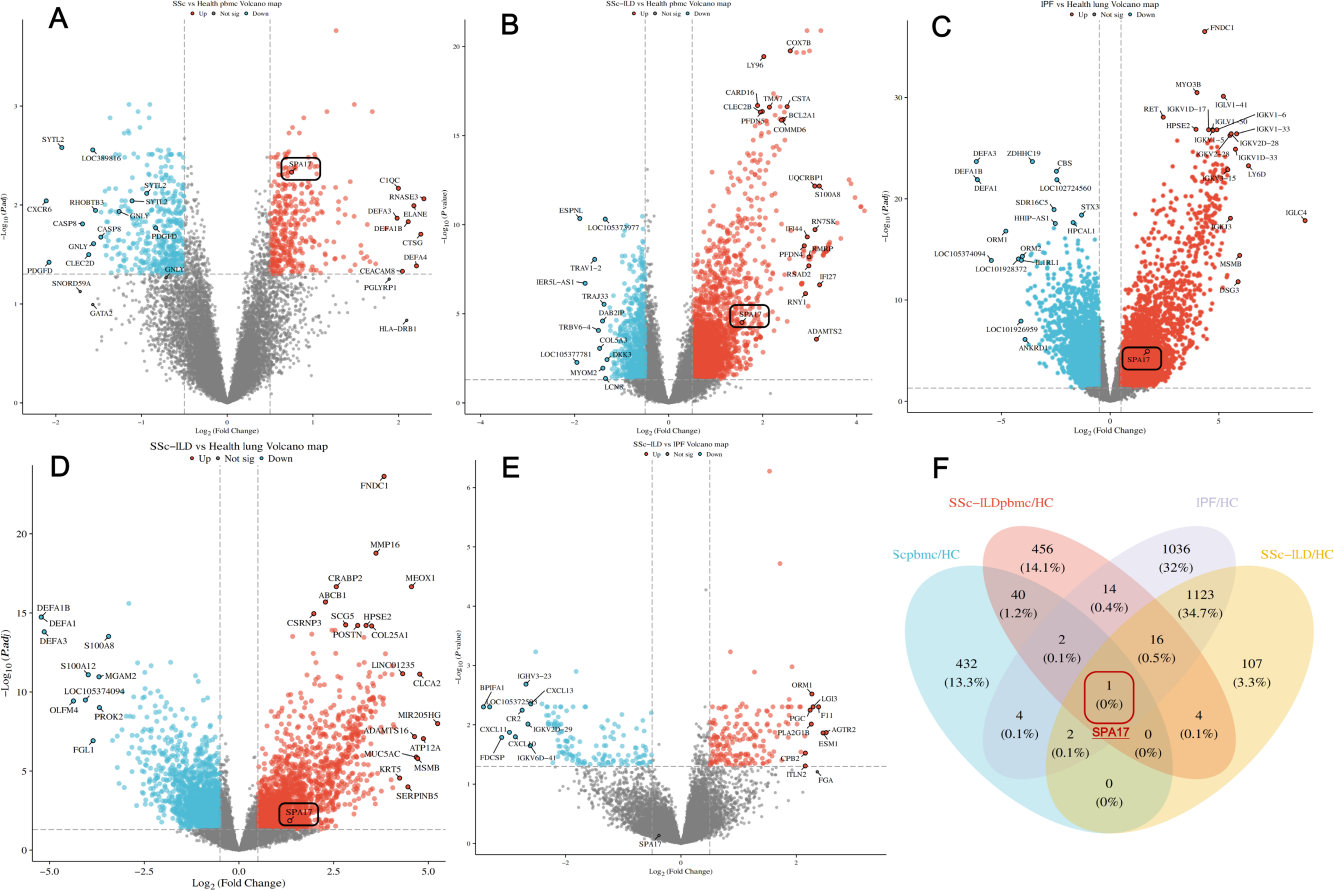
Volcano plot and Venn diagram. (A) Volcano plot: Deferential gene expression of PBMC among SSc patients and control. (B) Volcano plot: Deferential gene expression of PBMC among SSc‐ILD patients and controls. (C) Volcano plot: Deferential gene expression of lung among IPF patients and controls. (D) Volcano plot: Deferential gene expression of lung among SSc‐ILD patients and controls. (E) Volcano plot: Deferential gene expression of lung among SSc‐ILD patients and IPF patients. (F) Venn diagram: The intersection of upregulated genes among the four groups SSc versus control (pbmc), SSc‐ILD versus control (pbmc), IPF versus control (lung), and SSc‐ILD versus control (lung) was analyzed.

Compared with healthy controls, genes associated with inflammatory response and immune regulation, such as CTSG, RNASE2, RNASE3, DEFA4, SERPINB2, and ORM1, were markedly elevated among SSc patients' pbmc (Figure 6A; Supplementary File 3). In the peripheral blood of SSc‐ILD patients, upregulated genes including S100A8, S100A12, ADAMTS2, IFNG, and SERPINB2 were identified, which have been linked to the progression of lung fibrosis (Figure 6B; Supplementary Files 4). Interestingly, further exploration of transcriptomic differences between IPF patients and healthy controls revealed a significant presence of IgG antibody transcripts, such as IGKV1D‐17, IGLV1‐41, and IGKV2D‐28 (Figure 6C and Supplementary [Link], [Link], [Link]). In the lung tissues of SSc‐ILD patients, there was a notable increase in the expression of MMPs (e.g., MMP16), epithelial markers (e.g., KRT5, SPA17), and fibrosis‐related genes (e.g., COL25A1) (Figure 6D).

Enrichment analysis of these upregulated genes was conducted, with results showing that peripheral blood from SSc patients was enriched in pathways related to T cell migration (e.g., DEFA1B/DEFA1/ECM1, Padj = 0.0166) and mononuclear cell migration (e.g., CTSG/DEFA1B/DEFA1/C3AR1/ECM1, Padj = 0.0077).

Pathway enrichment analysis for SSc‐ILD patients' peripheral blood indicated significant enrichment in ECM‐receptor interaction pathways (e.g., TNN/COL4A6/THBS4/COL4A5/ITGB4/SPP1/COL9A2/COMP/COL9A3/SV2B/FREM1/ITGA11/COL1A1/ITGA7/GP5/RELN/COL1A2, Padj = 0.0022) and cytokine‐cytokine receptor interaction pathways (e.g., CXCL14/NGFR/CD27/CXCL13/CXCL12/TNFRSF13B/TNFRSF13C/TNFRSF19/TNFRSF17/IL31RA/GDF7/BMP7/CCL24/CCL14/BMP4/CXCL11/TNFRSF18/INHBB/IL13RA2/BMPR1B/EDAR/EDA2R/CXCL6/CXCL10/IL11/CNTFR/IL5RA/IFNG/CCR6/GDF6/CCL22/CCR7/CNTF/CCL21/CX3CL1/CCL16/CCL8, Padj = 0.0033). More details can be found in Supplementary [Link], [Link], [Link], [Link].

#### 3.2.4 Transcriptomic Data Reveal the Shared Upregulated Gene SPA17 in Peripheral Blood and Lung Tissue of SSc‐ILD Patients and Lung Tissue of IPF Patients

Given that both SSc‐ILD and IPF are classified as ILD, we were curious that if there were common upregulated genes among all of those groups or enriched pathways across the comparisons of SSc versus healthy controls (pbmc), SSc‐ILD versus healthy controls (pbmc), SSc‐ILD versus healthy controls (lung), and ILD versus healthy controls (lung). A Venn diagram analysis revealed that SPA17, a gene involved in cilium movement, was the common overlapping upregulated gene across all four groups (Figure 6F). Additionally, enriched pathways in the shared gene set included humoral immune response, collagen‐containing extracellular matrix, and leukocyte migration (Supplementary Files 12).

**FIGURE 7:**
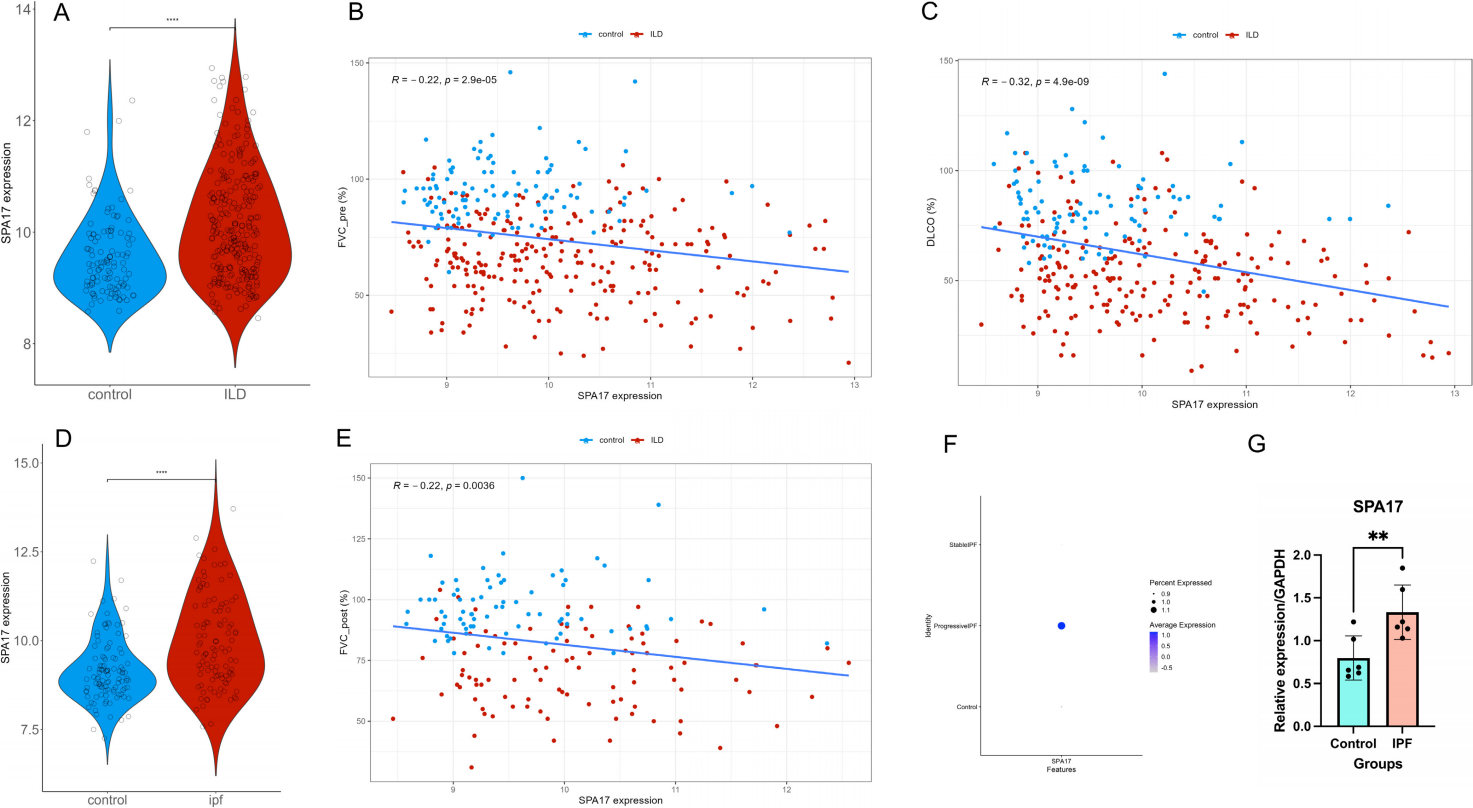
The relationship of SPA17 and lung functions. (A) Violin plot of SPA17 expression in GSE47460. (B) Linear regression analysis between individual lung function parameter FVC (pre) and lung SPA17 gene expression levels. (C) Linear regression analysis between individual lung function parameter DLCO and lung SPA17 gene expression levels. (D) Violin plot of SPA17 expression in GSE150910. (E) Linear regression analysis between individual lung function parameter FVC (pro) and lung SPA17 gene expression levels. (F) Dot plot of SPA17 gene expression in IPF single‐cell dataset, which group by different disease conditions. (G) SPA17 Q‐PCR validation experiment.

To further explore the potential relationship between SPA17 and clinical outcomes in pulmonary fibrosis patients, we downloaded data from GSE47460 (lung tissues from 254 IPF patients and 108 healthy controls) [23] and GSE150910 (lung tissues from 103 IPF patients and 103 healthy controls) [24]. The analysis indicated a significant association between SPA17 expression and the occurrence of IPF. Additionally, linear regression data demonstrated that SPA17 expression was negatively correlated with FVC‐pre, FVC‐pro, and DLCO, suggesting that elevated SPA17 levels may be linked to worse clinical outcomes in pulmonary fibrosis patients (Figure 7A–E). And also in the single‐cell dataset, SPA17 expressed higher among progressive IPF patients compared with control or stable IPF patients (Figure 7F).

#### 3.2.5 Human Sample Validation

Due to the relatively low number of SSc‐ILD patients visiting Beijing Chaoyang Hospital, we collected pbmcs from six IPF patients and six age‐ and sex‐matched control patients from the Beijing Chaoyang Hospital human sample repository for qPCR verification (clinical information of patients can be found in Supplementary File 13). The results showed that the SPA17/GAPDH ratio in the peripheral blood of IPF patients was significantly higher compared with the control group (0.6723 [0.6319–0.9349] vs 1.1903 [1.1441–1.5034], *p* = 0.0096; Figure 7G). The raw data can be found in Supplementary Files 14.

## 4 Discussion

### 4.1 Main Findings of the Study

In this study, we found that the CD4+/CD8+ T cell ratio of pbmc in SSc‐ILD patients was significantly higher than that in SSc‐nonILD patients. Similar observations were reflected in single‐cell sequencing of IPF patients' pbmc, where the CD4+/CD8+ T cell ratio in the peripheral blood of progressive IPF patients was significantly higher than that of control patients. And Treg and mature CD4+ T cells might cause this bias between different disease conditions, which suggesting that an imbalance in T cell subpopulation numbers and ratios may play a potential role in the development and progression of ILD. Although the differences of CD4+/CD8+ T cell ratio among SSc‐ILD lung tissues did not reach the statistical significance between SSc‐ILD and control groups, there was an increase trend that can be found among the SSc‐ILD groups versus the control group. In terms of changes of gene functions, IPF‐related differential genes in CD4+ and CD8+ T cells were predominantly enriched in the cytokine–cytokine pathway and JAK–STAT pathway.

While SSc‐ILD pbmc pathways enriched in ECM–receptor interaction pathways and cytokine–cytokine receptor interaction pathways. Additionally, the CD30 pathway (TNFSF8 and TNFRSF8), CD40 pathway (CD40LG, ITGA5, ITGAM, ITGB1, ITGB2), and FLT3 signaling pathway (FLT3LG and FLT3) were identified as primary pathways mediating communication between T cells and other immune cells in IPF patients' peripheral blood. Finally, transcriptomic data from SSc, SSc‐ILD, and IPF patients, combined with single‐cell sequencing of IPF and small‐cohort qPCR from peripheral blood, indicated that SPA17 may play as an pan‐marker in ILD and associate with deterioration of lung function.

### 4.2 Significance of the Study Findings

The diagnosis of SSc‐ILD and IPF has become relatively straightforward, yet despite aggressive pharmacological and supportive treatment, a portion of patients continue to experience significant deterioration in lung function. The CD4+/CD8+ T cell ratio was initially proposed during the treatment of organ transplantation and HIV patients [25, 26] where the imbalance between CD4+ and CD8+ T cell numbers indicated disturbances in humoral and cellular immunity within patients. With the growing understanding of ILD, the functional abnormalities of CD4+ and CD8+ T cell subgroups have been increasingly emphasized in the progression of ILD [5, 27, 28]. For instance, the bronchoalveolar lavage fluid (BAL) of patients with sarcoidosis often shows an elevated CD4+/CD8+ T cell ratio [29]. An increased CD4+/CD8+ T cell ratio suggests heightened cytotoxic activity and accelerated cellular destruction and has been associated with the progression of ILD in patients with anti‐MDA5 antibodies [18]. Beyond the numerical differences in T cell ratios, the genetic and functional variations within T cells warrant further exploration. Tregs are not only elevated in pbmcs but have also increased in the lungs of patients with SSc‐ILD compared with control groups; research suggests that disease activity may be associated with an increased number of Tregs [28, 30]. Additionally, the ratio of Tim‐3+, ICOS+, and OX40+ in CD4+ T cells is significantly higher in patients with CT features of usual interstitial pneumonia compared with the non‐ILD group, which aligns with the findings of this study [31].

Through public single‐cell sequencing data analysis of IPF, we found significant activation of the JAK–STAT and cytokine–cytokine pathways in aberrant T cells, particularly in Naive CD8 T cells, consistent with previous studies [32]. Additionally, the CD30 pathway (TNFSF8 and TNFRSF8), CD40 pathway (CD40LG, ITGA5, ITGAM, ITGB1, ITGB2), and FLT3 signaling pathway (FLT3LG and FLT3) played significant roles in immune regulation of T cells in ILD patients. TNFSF and TNFRSF molecules are widely present in all mammals and are highly conserved through evolution [33, 34]. It has been demonstrated that TNF, a pro‐inflammatory factor, and anti‐TNF antibodies or Fc fusion proteins (TNF blockers) have shown remarkable efficacy in autoimmune‐related diseases such as rheumatoid arthritis and Crohn's disease [33, 34]. TNFSF8 stimulation of TNFRSF8‐expressing cells triggers diverse cellular signaling responses, including proliferation, survival, cytokine secretion, and apoptosis [35]. Although research on TNFSF8 and TNFRSF8 is limited, existing studies suggest their elevation may be associated with chronic inflammatory diseases, including lupus erythematosus, asthma, rheumatoid arthritis, and atopic dermatitis [36]. Similarly, studies on the CD40 pathway (CD40LG, ITGA5, ITGAM, ITGB1, ITGB2) within the T cell‐monocyte‐DC‐NK axis in ILD patients remain sparse. However, human and murine models of atherosclerosis indicate that the CD40L‐CD40 interaction in T cells and dendritic cells is critical for interferon‐γ production [37]. Elevated integrin α5 expression in fibroblasts derived from IPF is linked to paracrine TNF‐α signaling, potentially contributing to IPF pathogenesis [38]. ITGAM promotes macrophage differentiation into the M2 subtype, a key cell population in the development of lung fibrosis [39]. ITGB1, considered a marker of renal fibrosis, can be targeted with miR‐124‐3p to reverse fibrosis progression [40, 41], and its inhibition has shown potential in mitigating silicosis severity in mice [42]. ITGB2 is upregulated in the peripheral blood and skin of SSc patients [43, 44], correlating positively with liver fibrosis [45] and post‐COVID‐19 lung complications [46]. Additionally, FLT3LG and FLT3 have been implicated in kidney and splenic fibrosis in mouse models [47, 48], although their connection to lung fibrosis remains underexplored. Overall, these findings reinforce the significant role of T cells in the development and progression of ILD, exhibiting distinct disease phenotypes in terms of both quantity and function.

SPA17, as another important finding in this study, has been identified as a predictor of poor clinical outcomes in malignancies such as breast cancer and as an effective immunotherapy response predictor in tumor patients [49, 50, 51]. In human lungs, SPA17 is highly expressed in ciliated columnar epithelial cells [52], with its functions often associated with the structure and movement of cilia. Surprisingly, in both SSc, SSc‐ILD, and IPF patients, regardless of whether in peripheral blood or lung tissue, elevated SPA17 expression was observed in disease groups, correlating positively with disease severity. This intriguing finding raises the question of whether genes previously considered to have specific functions may possess unexplored and common physiological and pathological roles among various ILD. In ILD patients, inflammation triggers continuous repair and tissue regeneration of endothelial and epithelial cells, and some cases involve distal airway dilation, potentially leading to the hypermetabolism and proliferation of ciliated columnar epithelial cells in the lungs, thereby increasing SPA17 expression. However, the cause of elevated SPA17 in peripheral blood remains unclear. It is possible that SPA17 affects the cytoskeleton of immune cells, influencing their migration and other functions.

### 4.3 Study Strength and Limitations

To date, this study is the first to focus on the CD4/CD8 T cell ratio of pbmc in patients with SSc‐ILD and SSc‐nonILD. Furthermore, besides clinical data, single‐cell data was used as another validation dataset to access the CD4/CD8 T cell ratio among various severity of IPF patients to make our result more robust and reliable. Moreover, this study not only find the value of CD4/CD8 T cell ratio among ILD patients but also mentioned that this might cause by the Treg and mature CD4+ T cells in the disease group. At the same time, this study is based on peripheral blood data collected from patients who visited Beijing Chaoyang Hospital over the past 13 years, making the dataset particularly valuable. Lastly, this study provides a more detailed examination of the gene functional characteristics of CD4+ and CD8+ T cells in the peripheral blood of ILD patients, especially those with IPF, and explores potential cell talk pathways through which these cells may communicate with other immune cells. These findings offer important insights and guidance for future studies into the immunological characteristics of SSc‐ILD in peripheral blood. However, limitations remain. The clinical data were sourced from a single center in Beijing, which may introduce selection bias. And the small sample size of q‐PCR limits the reliability of this conclusion. Additionally, the lack of high‐quality, disease‐related pbmc data for SSc‐ILD currently limits the ability to conduct detailed single‐cell analysis to gain a deeper understanding of peripheral blood immune regulation in SSc‐ILD patients.

### 4.4 Future Research Directions

Further analysis of the similarities and differences in immune responses between SSc‐ILD and IPF is necessary, and comprehensive cross‐sectional comparisons of lung and pbmc data using large datasets are urgently required. High‐quality single‐cell data on SSc‐ILD peripheral blood is strongly needed to fill research gaps. Finally, the reason for SPA17 elevation in the peripheral blood of ILD patients remains to be clarified by future functional studies.

## 5 Conclusion

The CD4+/CD8+ T cell ratio correlates with the occurrence and severity of ILD; increase Treg cells and mature CD4 T cells of disease group might cause this change. Abnormal T cells communicate with other immune cells in peripheral blood through CD30 (TNFSF8 and TNFRSF8), CD40 (CD40LG, ITGA5, ITGAM, ITGB1, ITGB2), and FLT3 (FLT3LG and FLT3) signaling pathways. SPA17 may be a potential predictive marker for ILD and is associated with lung function deterioration.

## Author Contributions

Conceptualization: Shuai Shao. Data curation: Shuai Shao, Yusha Chen and Zhijin Zhang. Formal analysis: Shuai Shao. Funding acquisition: Zhaohui Tong. Investigation: Shuai Shao. Methodology: Shuai Shao and Zhijin Zhang. Project administration: Zhaohui Tong. Resources: Shuai Shao and Siyu Cao. Software: Shuai Shao and Siyu Cao. Supervision: Yusha Chen and Zhaohui Tong. Validation: Shuai Shao. Writing – original draft: Shuai Shao and Siyu Cao. Writing – review and editing: Zhijin Zhang and Zhaohui Tong.

## Ethics Statement

This study was approved by the Research Ethics Board of Beijing Chao‐Yang Hospital (project approval number: 2023‐ke‐355). Informed consent was waived by the Research Ethics Board of Beijing Chao‐Yang Hospital.

## Consent for Publication.

Informed consent was waived due to the anonymous and mandatory nature of the data.

## Conflicts of Interest

The authors declare no conflicts of interest.

## Supporting information


**Supplementary Files 1** 1. CD4+ T cell and CD8 T cell count among IPF single‐cell Seurat project; 2.CD4+ T cell and CD8 T cell count among SSc‐ILD lung Seurat project; 3. Shapiro–Wilk test results of continuous variables in Table 1; 4. Shapiro–Wilk test results of cell proportions from IPF PBMC single‐cell dataset on disease level (control and IPF); 5. Shapiro–Wilk test for cell proportions from IPF PBMC single‐cell dataset on condition level (control, Stable IPF, progressive IPF); 6. Shapiro–Wilk test for cell proportions from SSc ILD lung single‐cell dataset on disease level (health and SSc ILD); 7. Multivariable analysis for CD4/CD8 T cell ratio.

**Supplementary Files 2** Treg KEGG analysis result, Cell–Cell talk among each cell type and CD 30, CD 40, and FLT3 pathways and related genes expression among different cell types.

**Supplementary Files 3** Deferential gene expression of PBMC among SSc patients and controls.

**Supplementary Files 4** Deferential gene expression of PBMC among SSc ‐ILD patients and controls.

**Supplementary Files 5** Deferential gene expression of lung among IPF patients and controls.

**Supplementary Files 6** Deferential gene expression of lung among SSc‐ILD patients and controls.

**Supplementary Files 7** Deferential gene expression of lung among SSc ‐ILD patients and IPF patients.

**Supplementary Files 8** KEGG pathway analysis of upregulated deferential genes of SSc versus controls (pbmc).

**Supplementary Files 9** KEGG pathway analysis of upregulated deferential genes of SSc‐ILD versus controls (pbmc).

**Supplementary Files 10** KEGG pathway analysis of upregulated deferential genes of IPF versus controls (lung).

**Supplementary Files 11** KEGG pathway analysis of upregulated deferential genes of SSc‐ILD versus controls (lung).

**Supplementary Files 12** Venn plot: The intersection of pathways of upregulated genes among the four groups SSc versus control (pbm), SSc‐ILD versus control (pbmc), IPF versus control (lung), and SSc‐ILD versus control (lung) was analyzed.

**Supplementary Files 13** Basic clinical data of samples used for Q‐PCR.

**Supplementary Files 14** The original data of Q‐PCR.

## Data Availability

The data that support the findings of this study are available on request from the corresponding author. The data are not publicly available due to privacy or ethical restrictions.
