# Supplementary figures and images for: Immunological Features and Potential Biomarkers of Systemic Sclerosis–Associated Interstitial Lung Disease and Idiopathic Pulmonary Fibrosis
Source: Clin Respir J. 2025 Mar 31;19(4):e70072. doi: 10.1111/crj.70072 (PMC11959098; doi:10.1111/crj.70072)

# Top 10 KEGG Pathways Enriched for Treg cells

KEGG Pathway

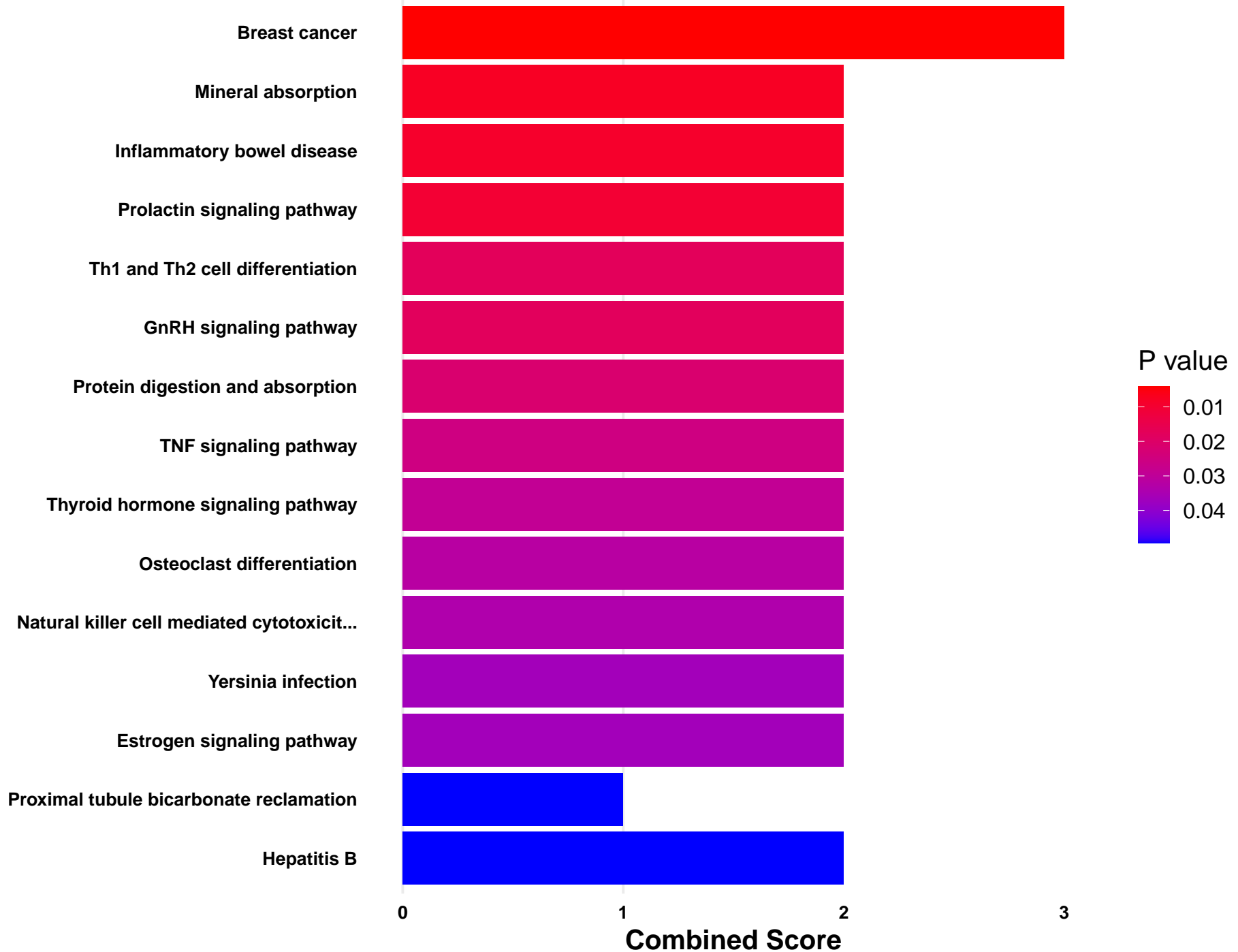

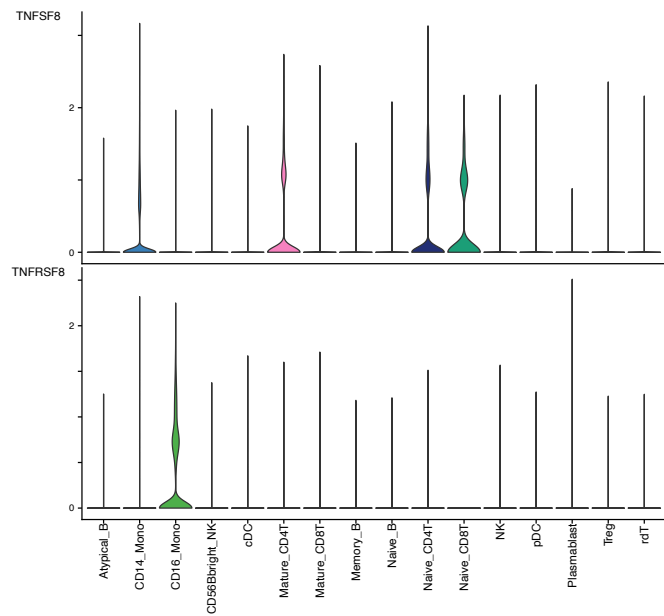

CD 30 pathway

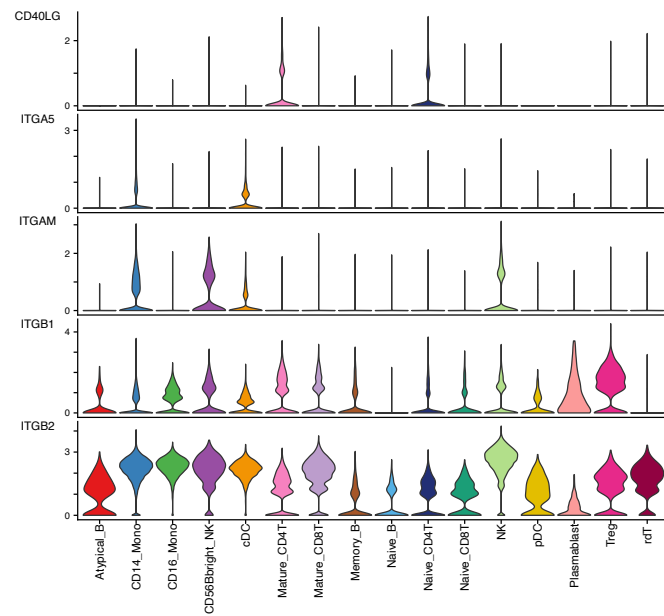

CD 40 pathway

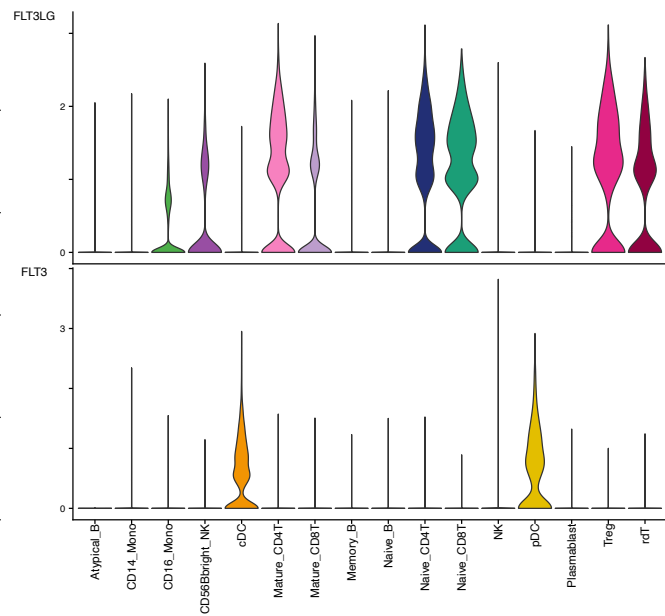

FLT3 pathway

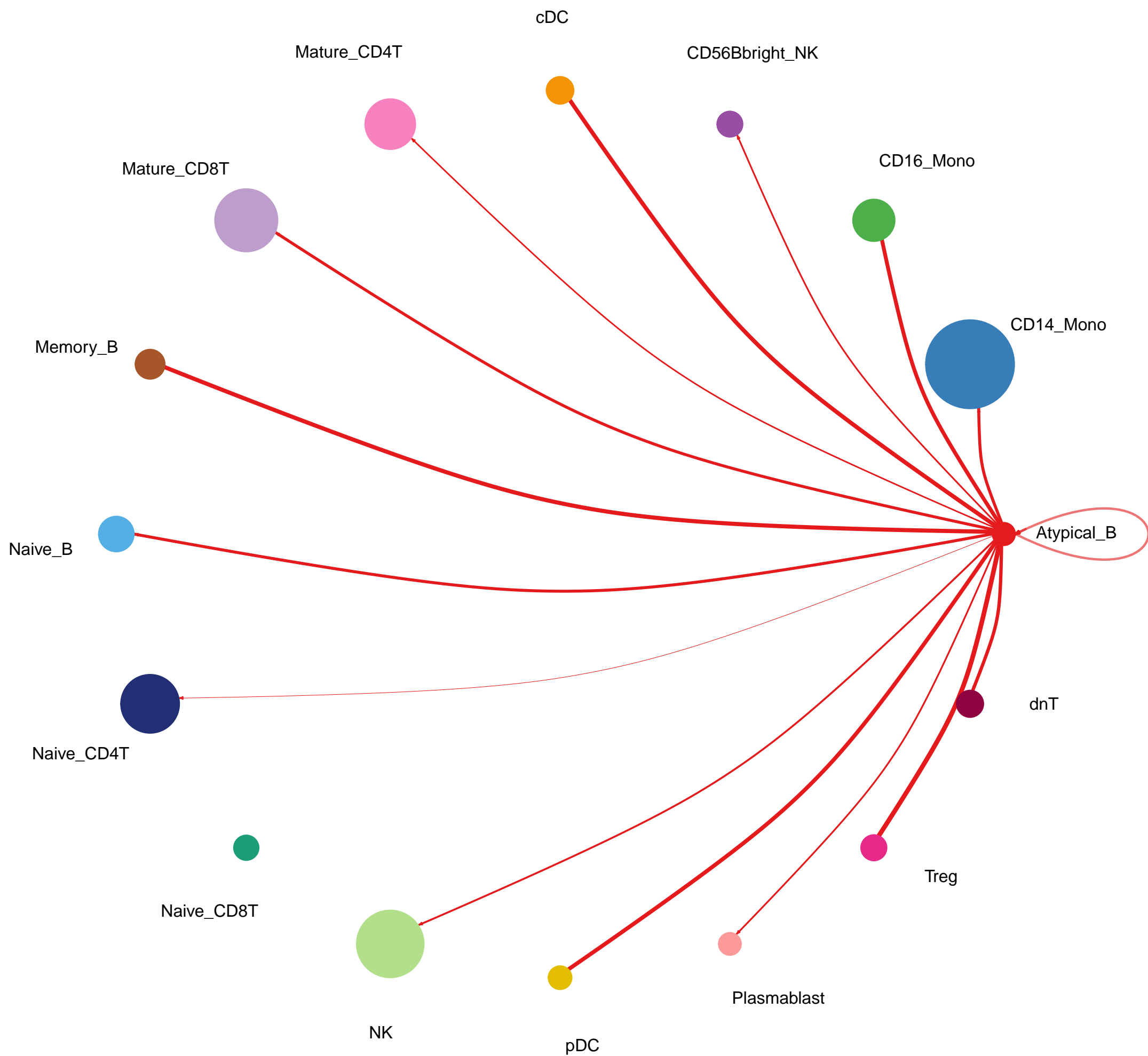

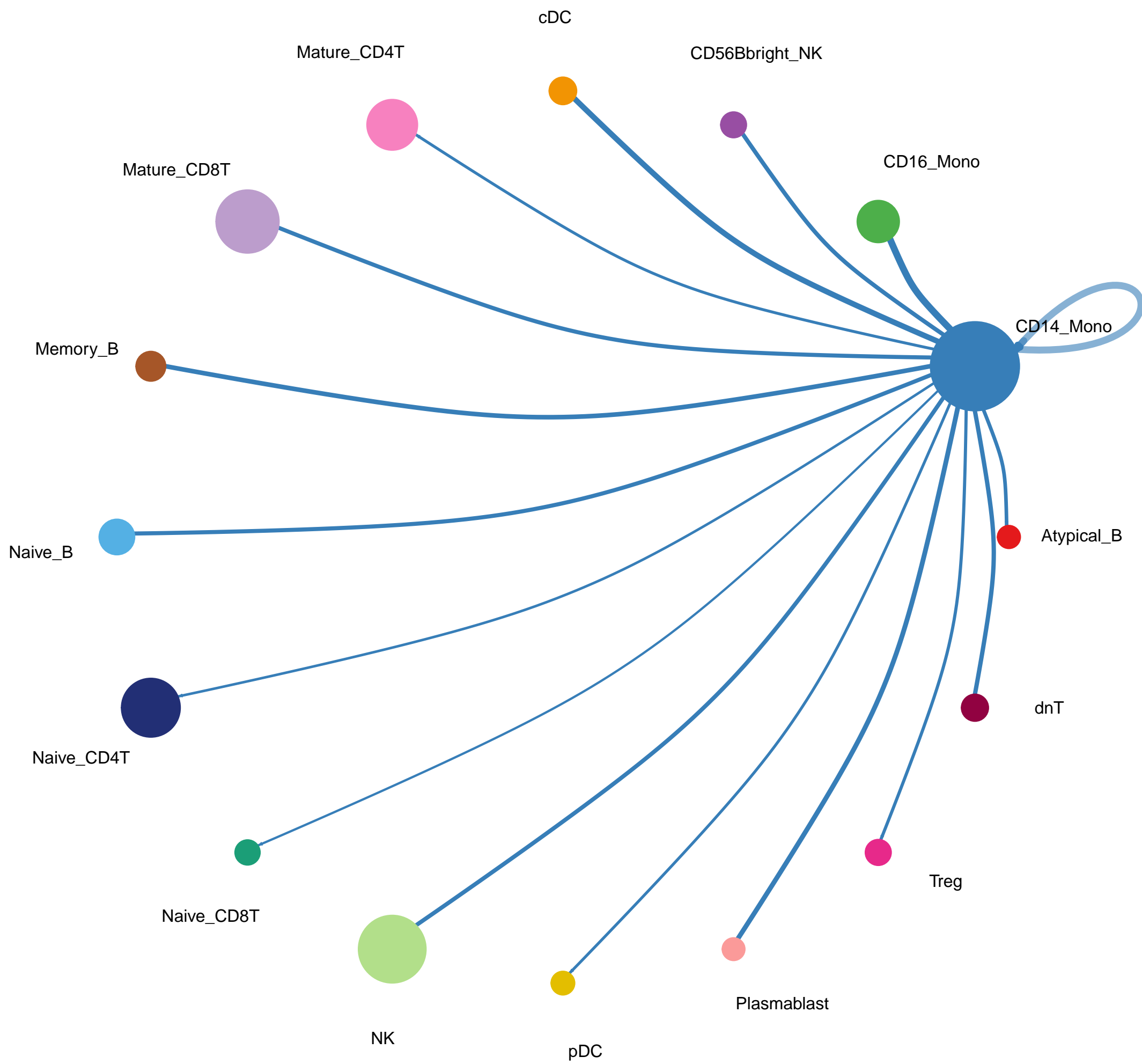

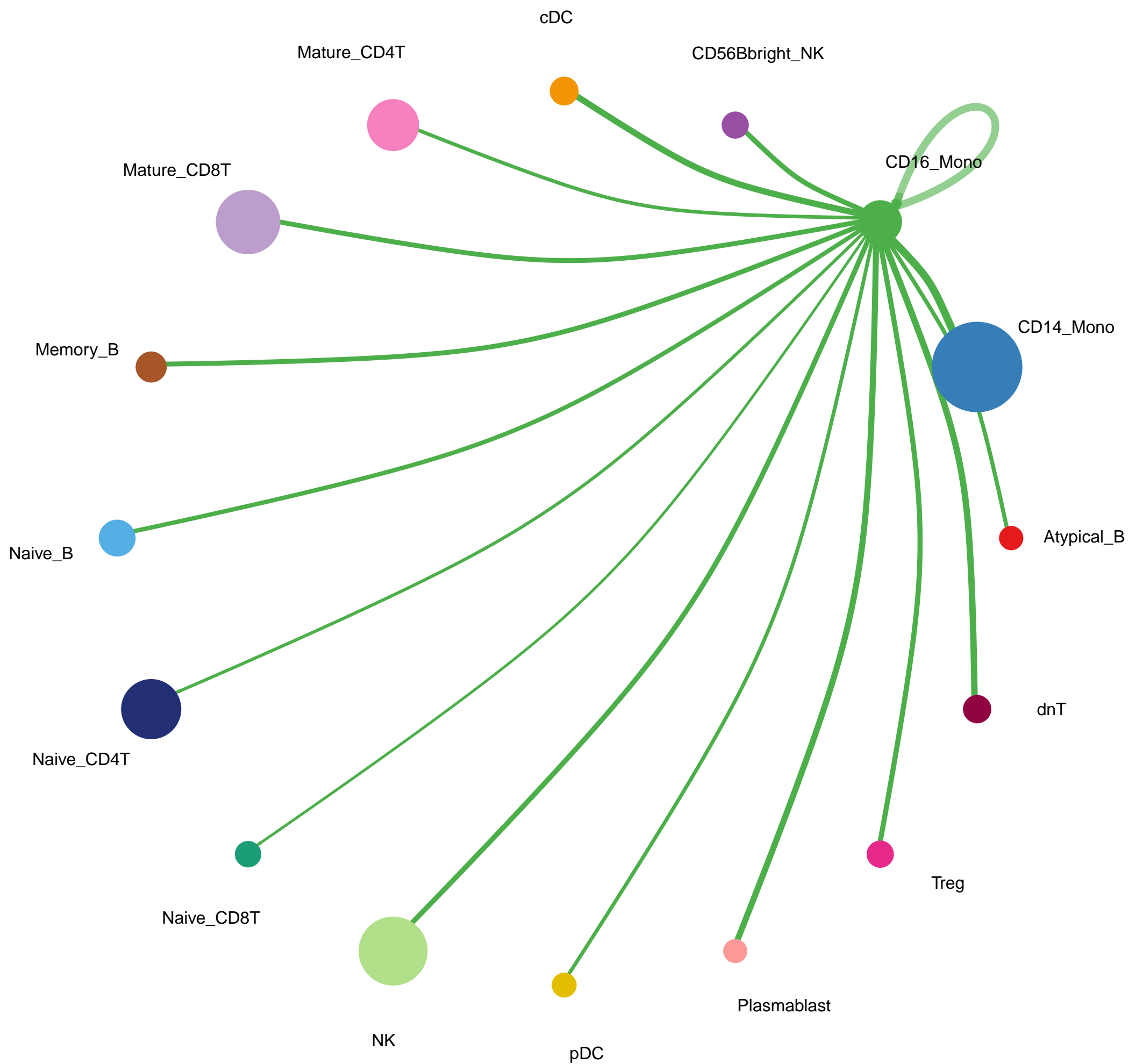

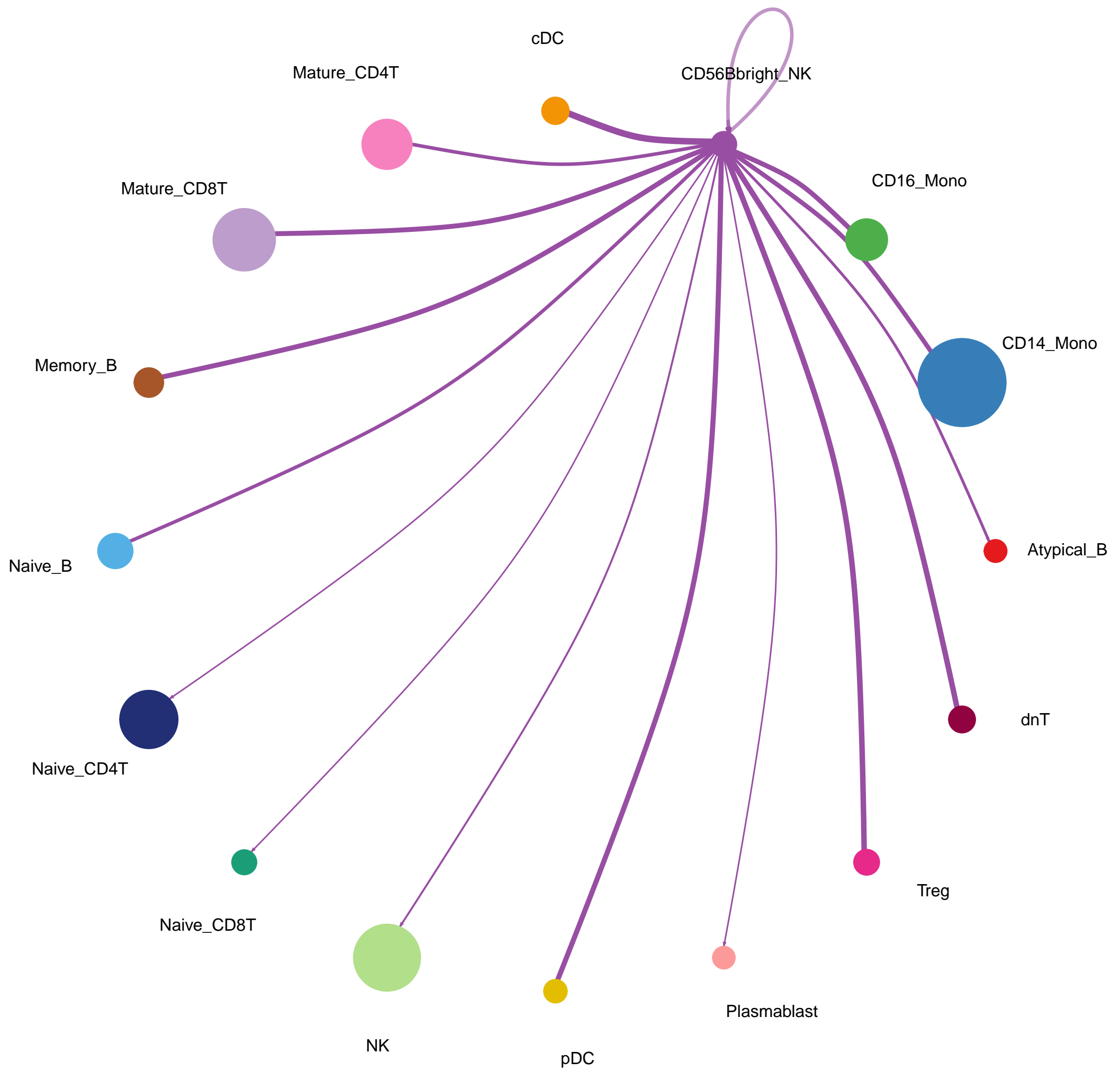

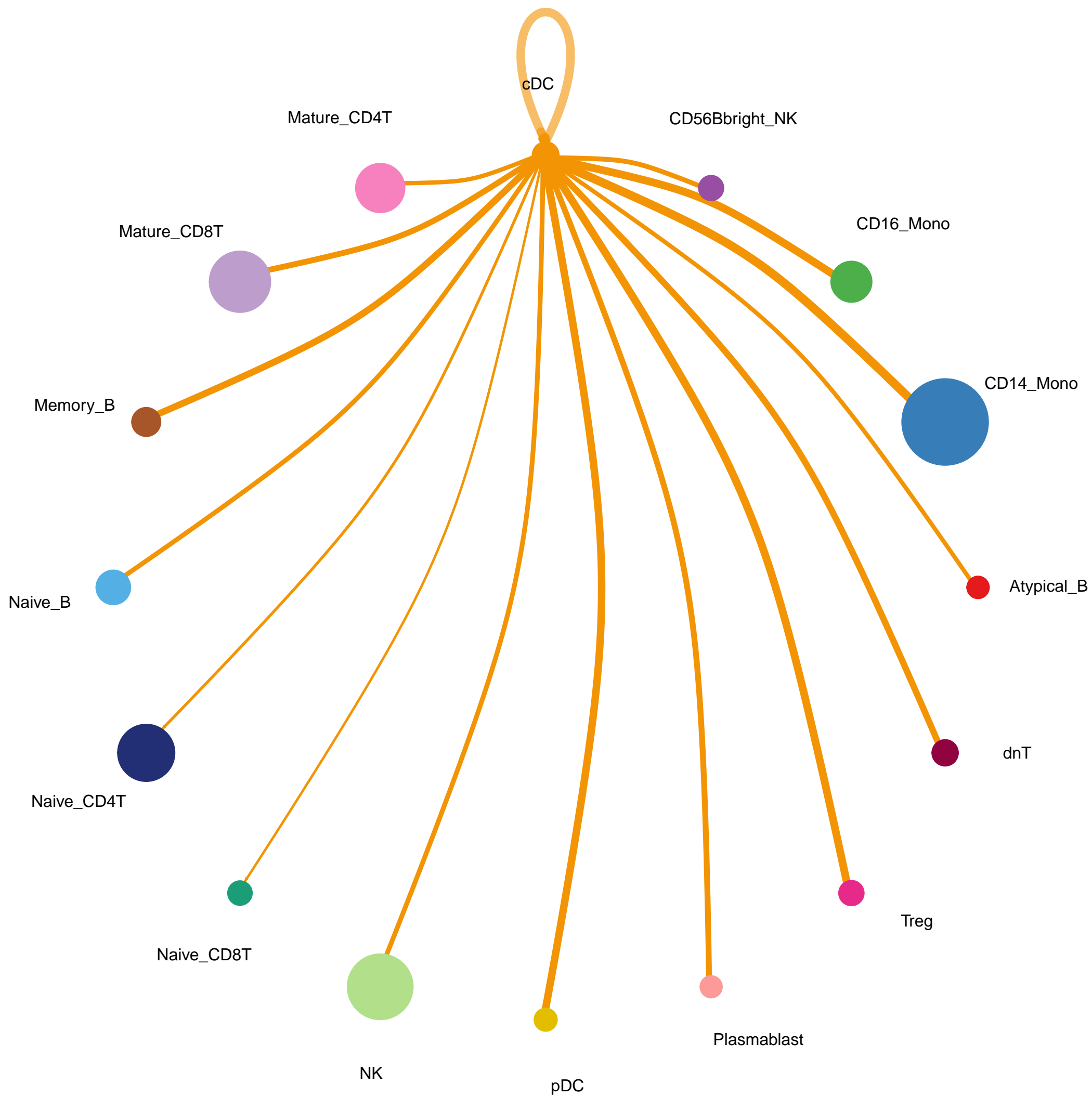

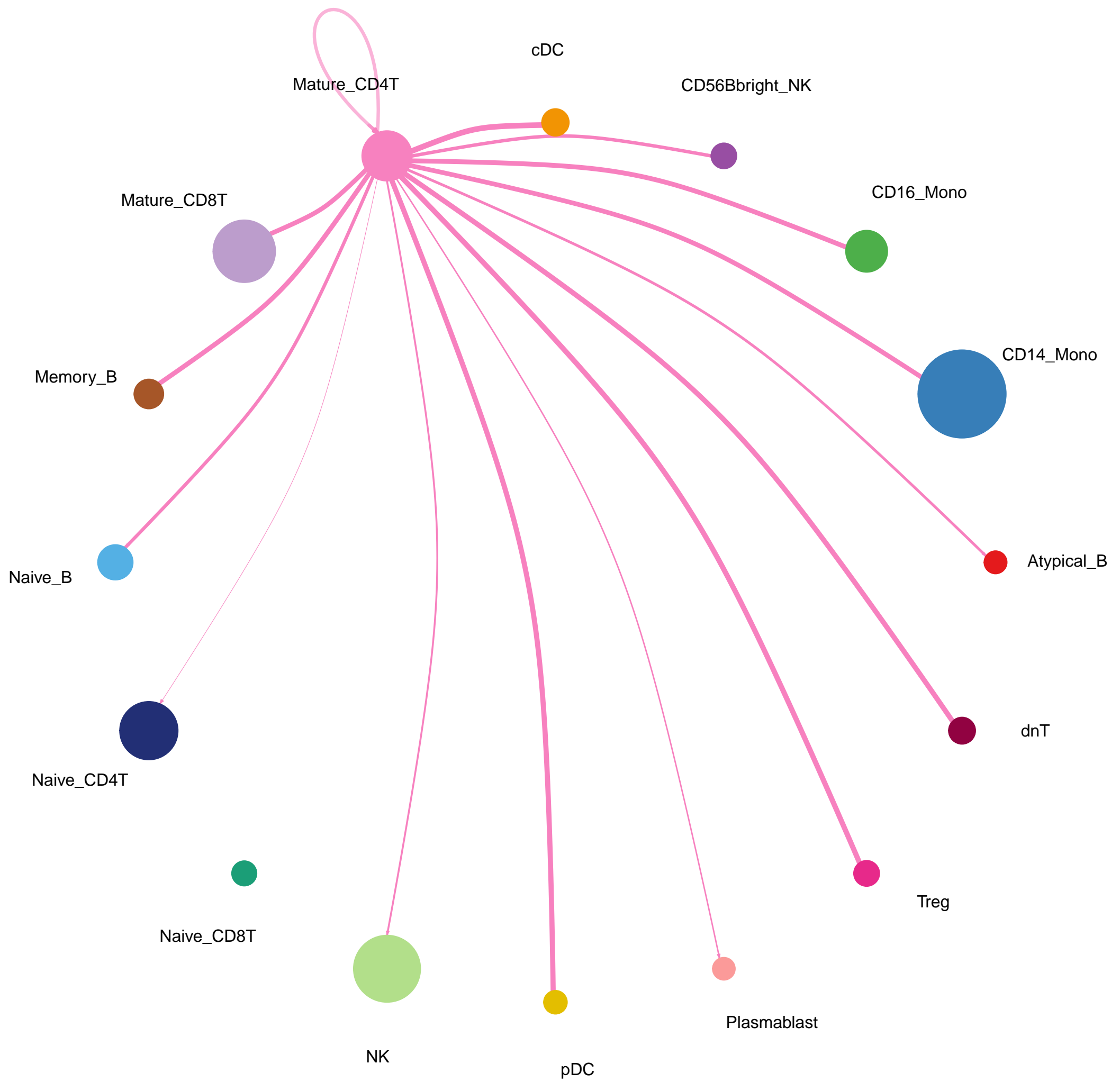

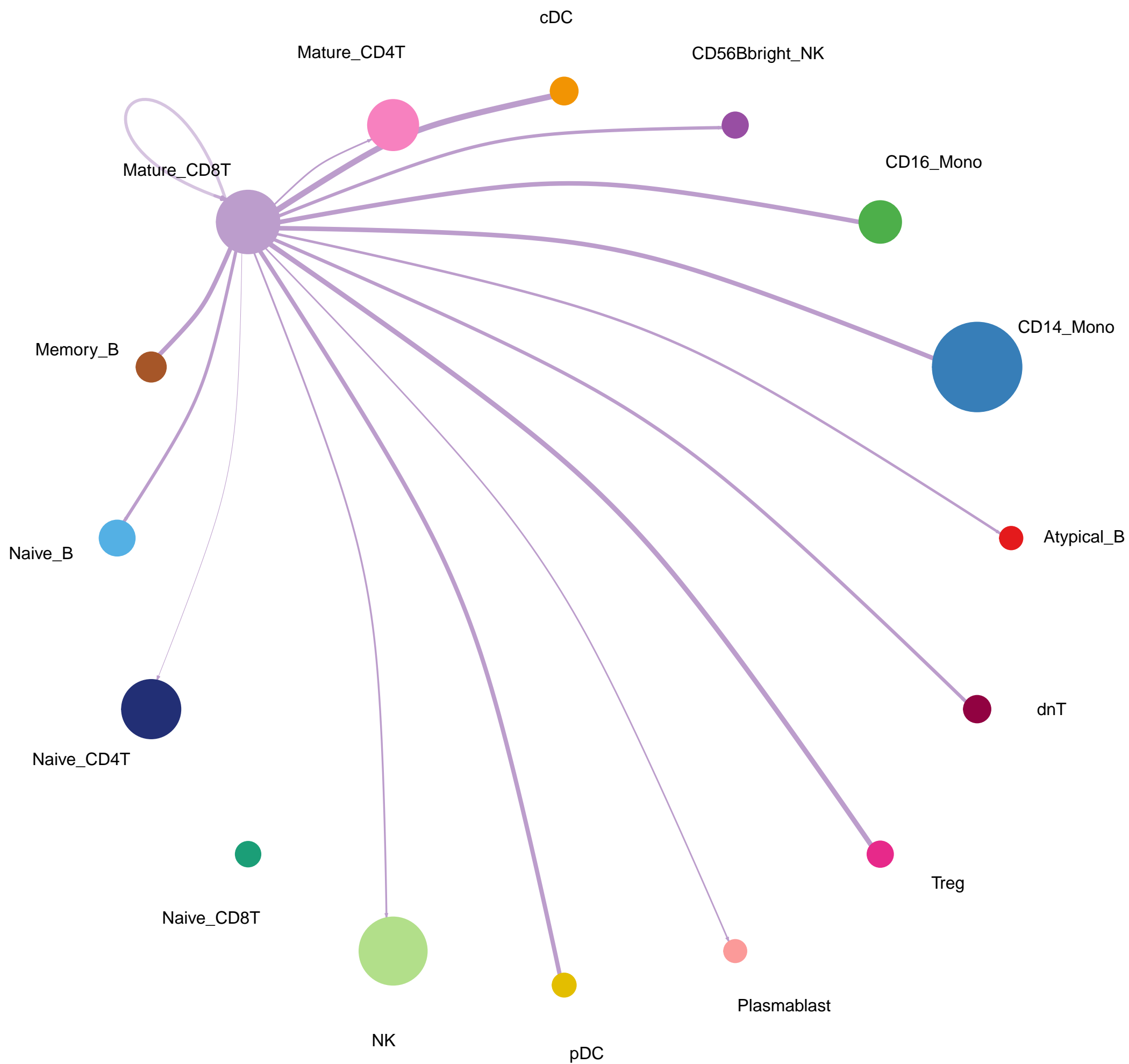

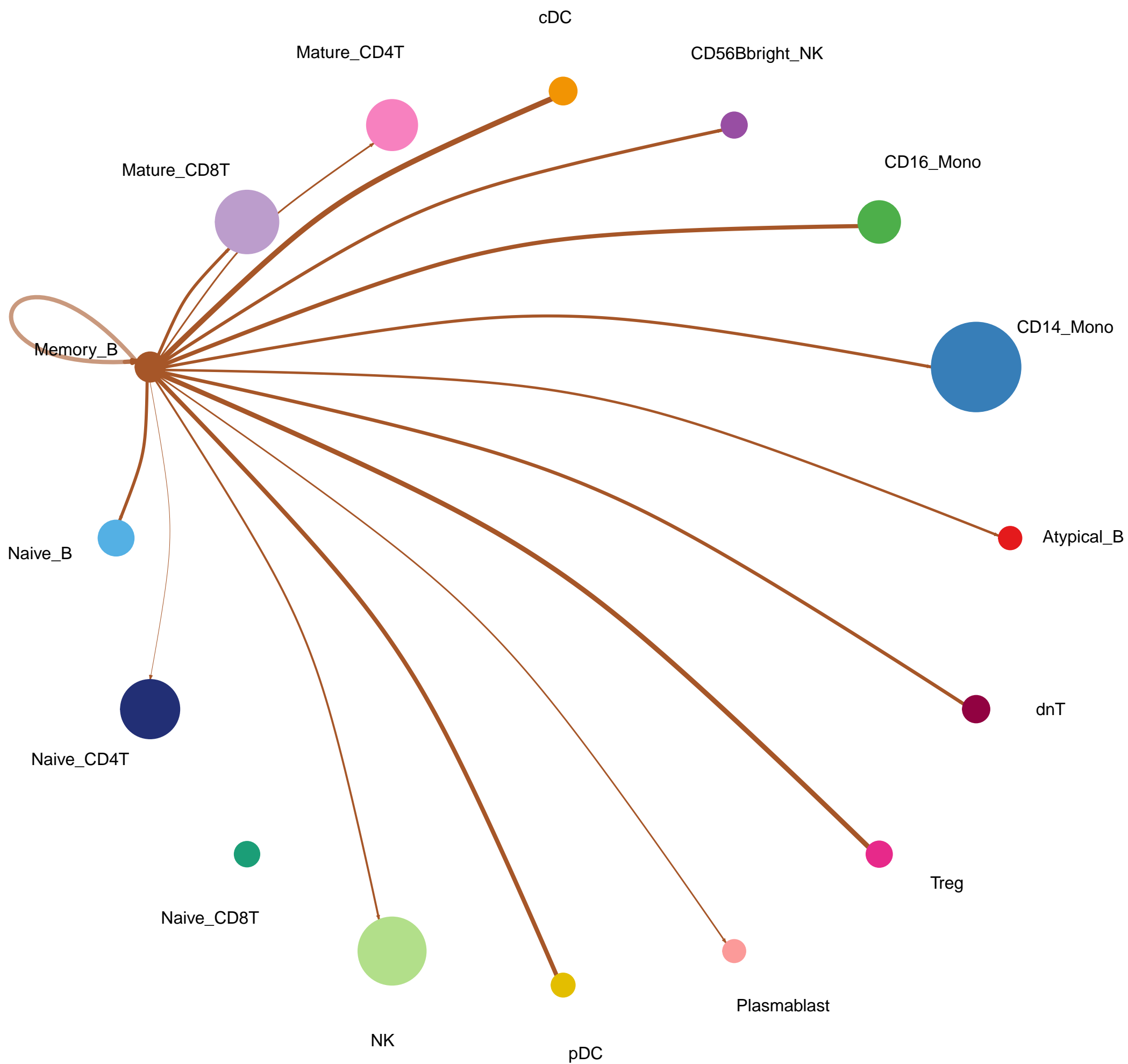

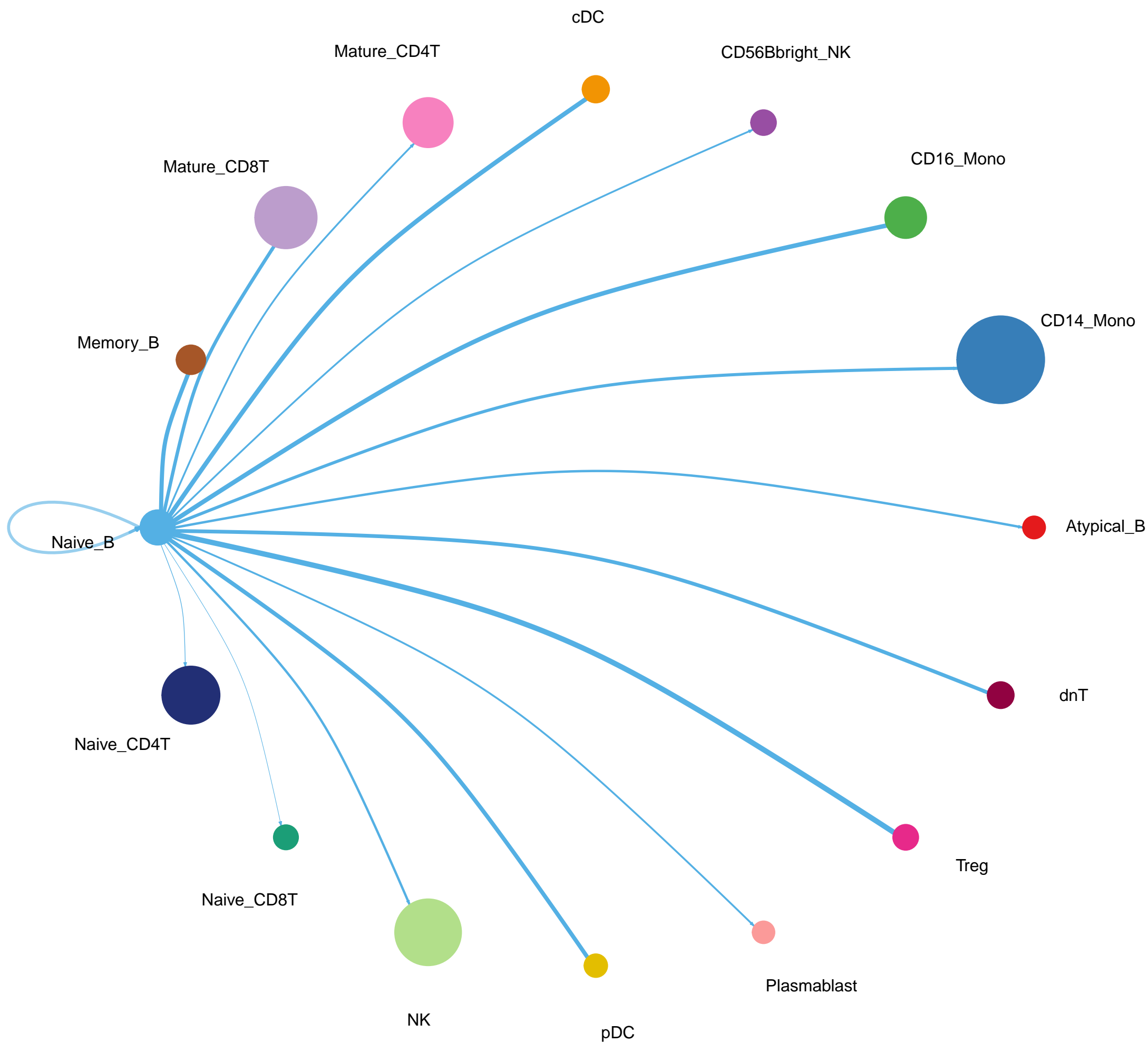

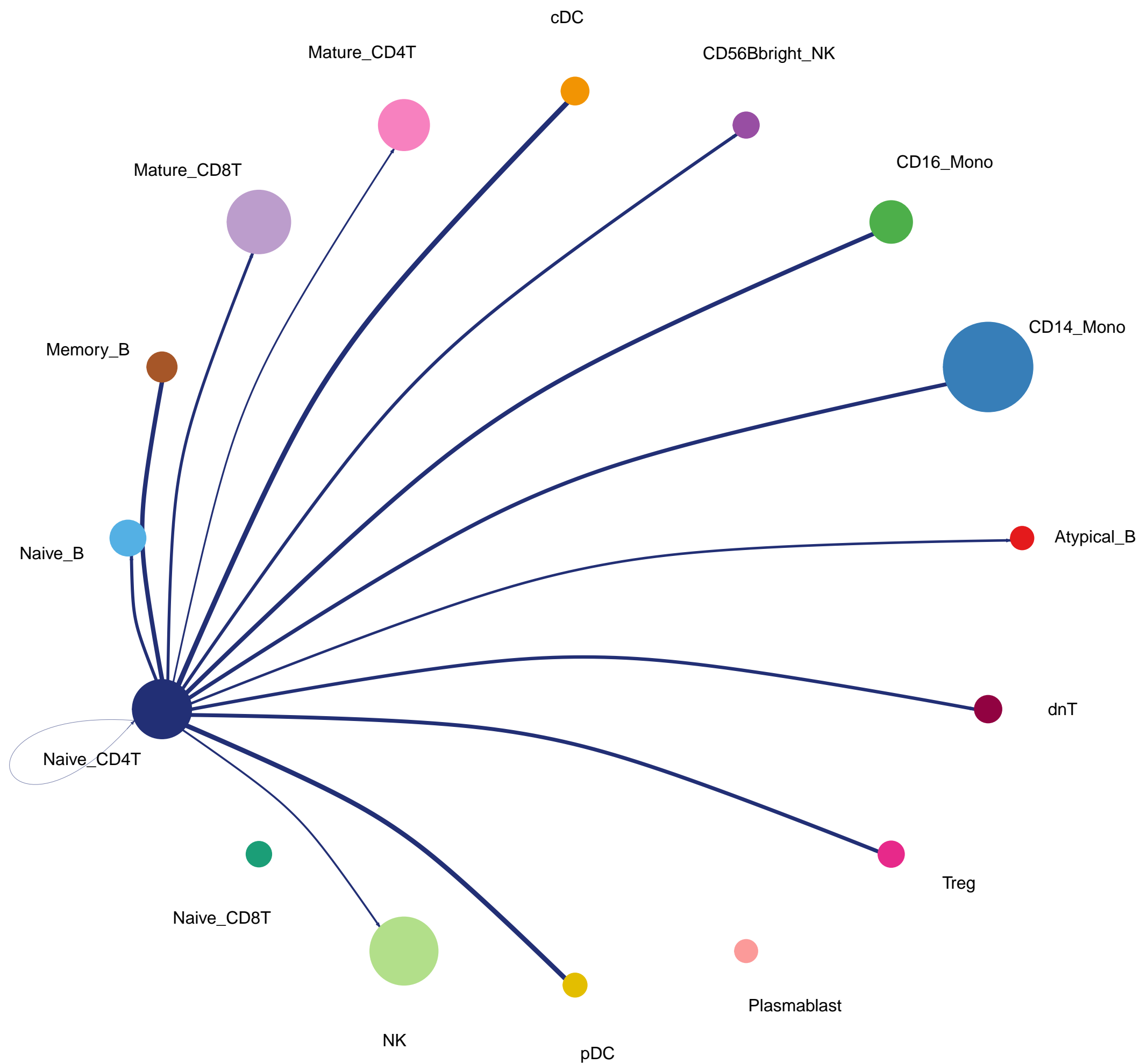

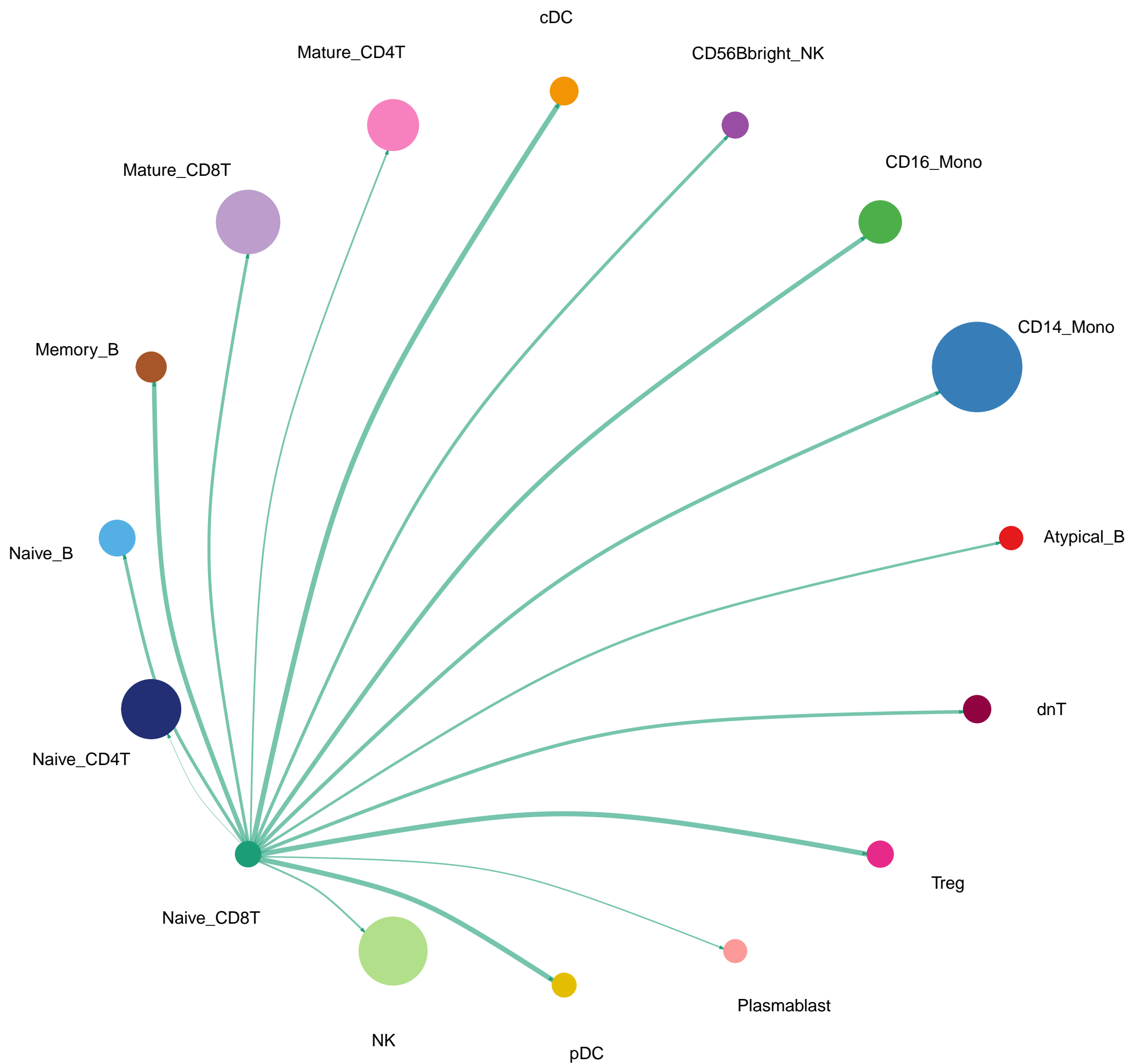

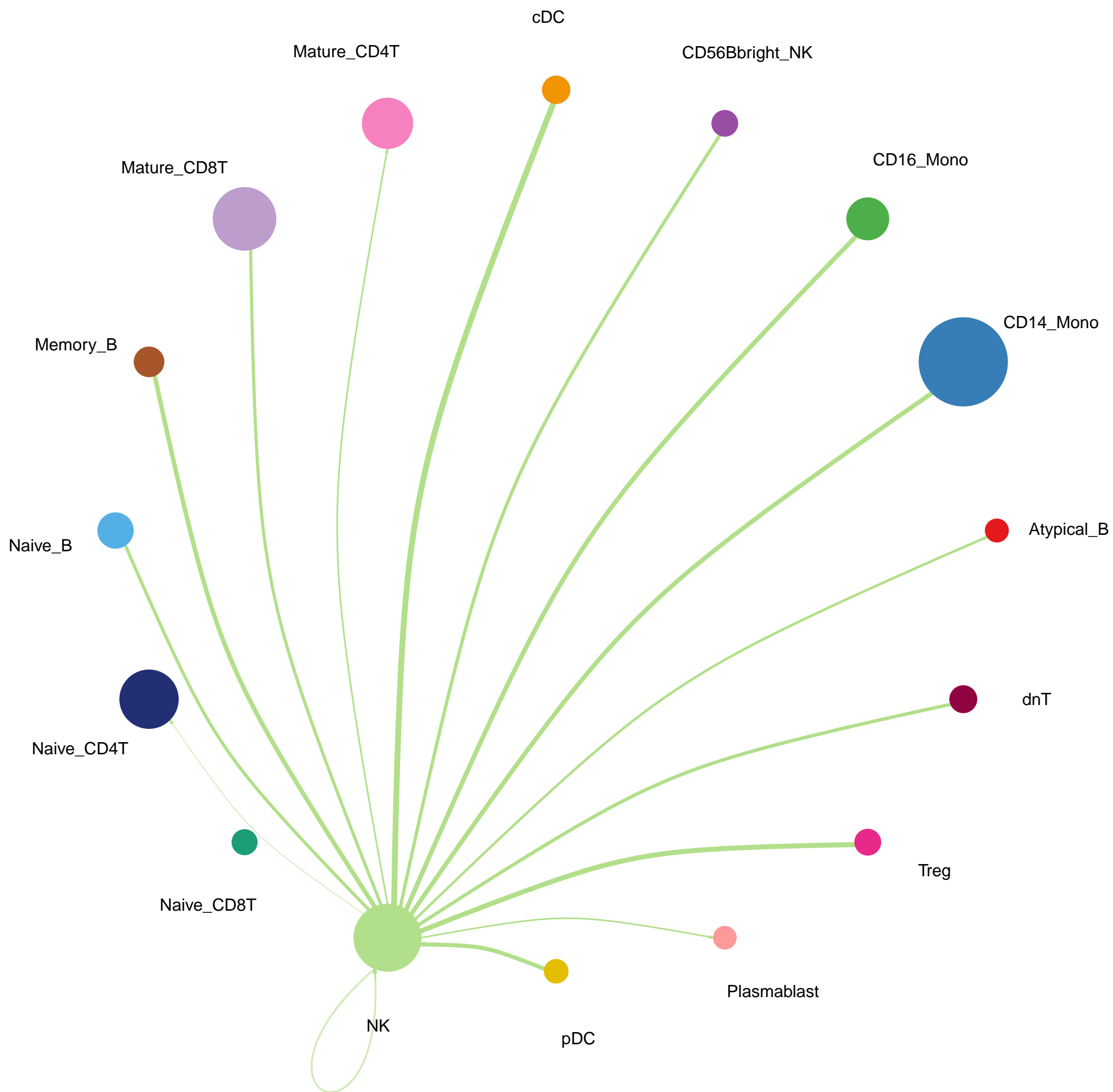

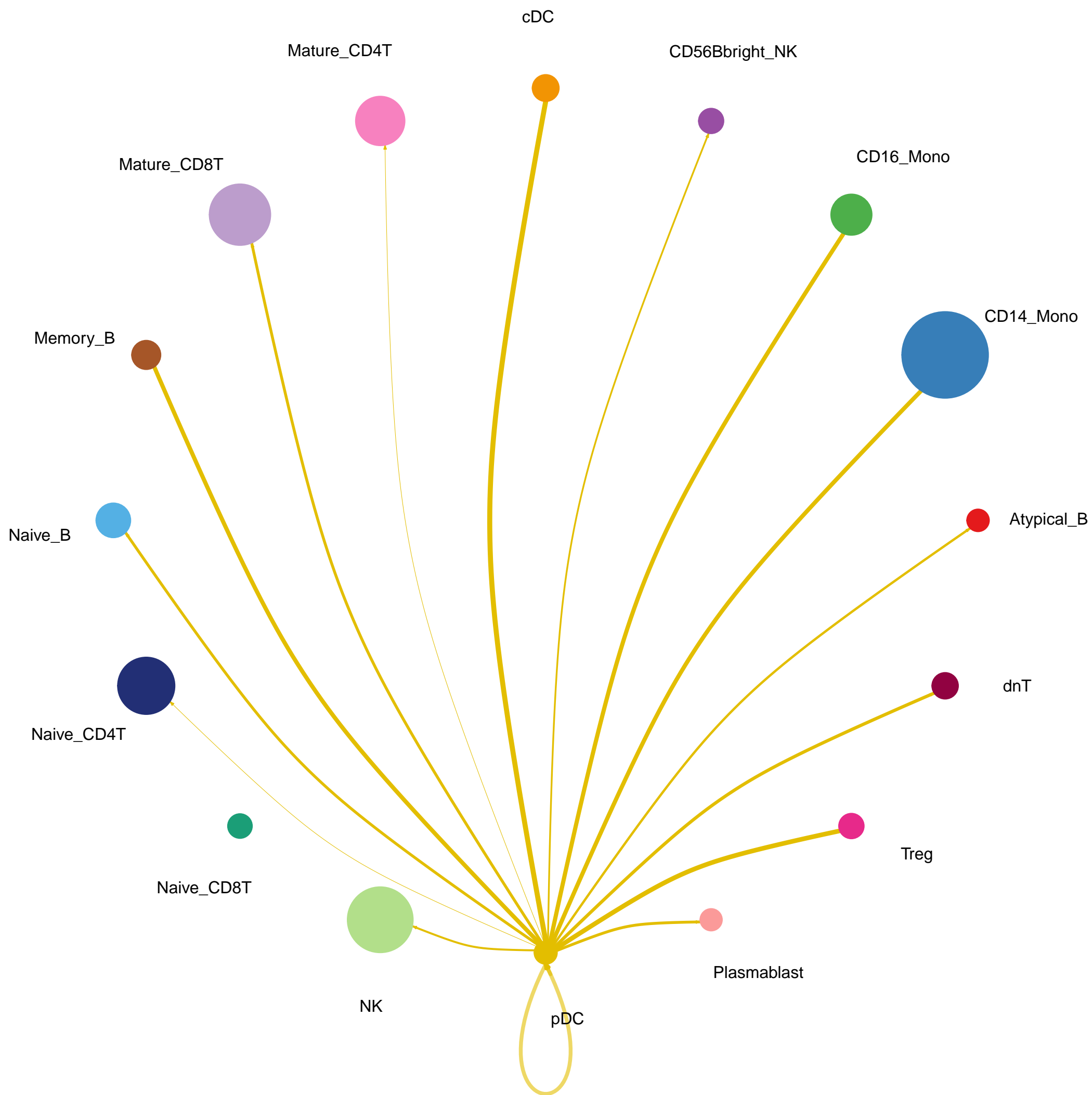

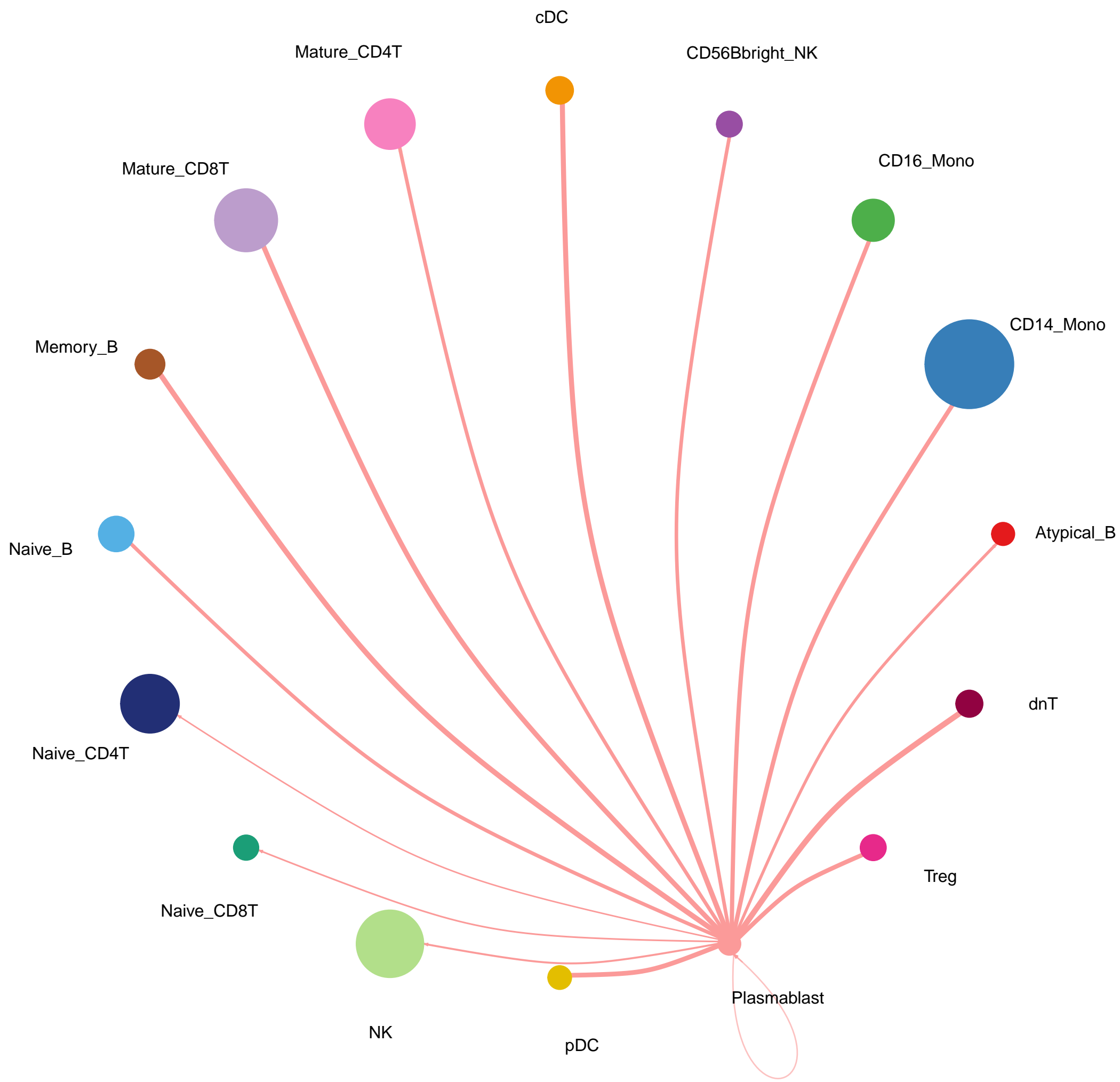

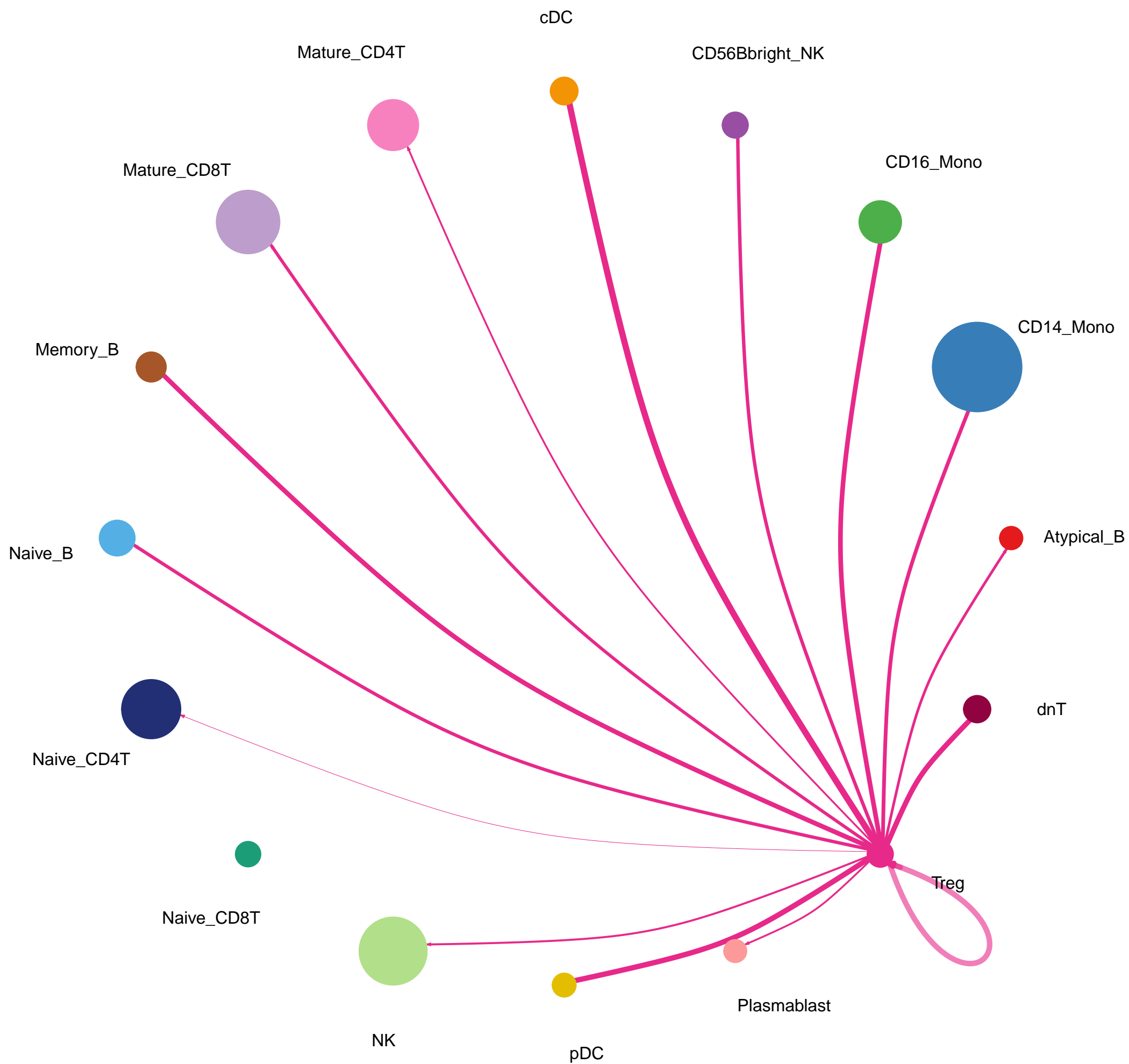

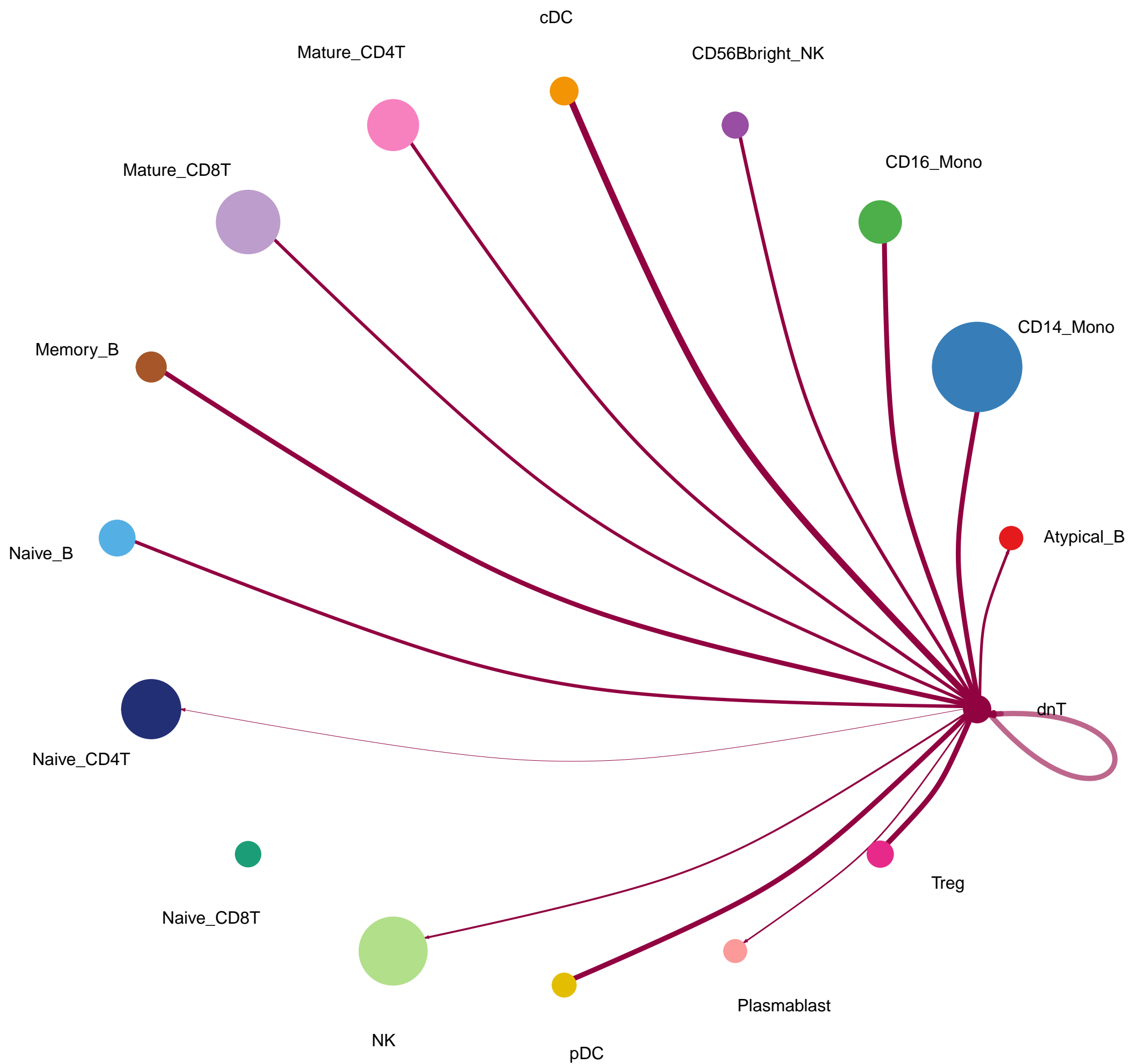

Supplement: Supplementary file 2 — Supplementary Files 2 Treg KEGG analysis result, Cell–Cell talk among each cell type and CD 30, CD 40, and FLT3 pathways and related genes expression among different cell types. [file CRJ-19-e70072-s010.pdf]

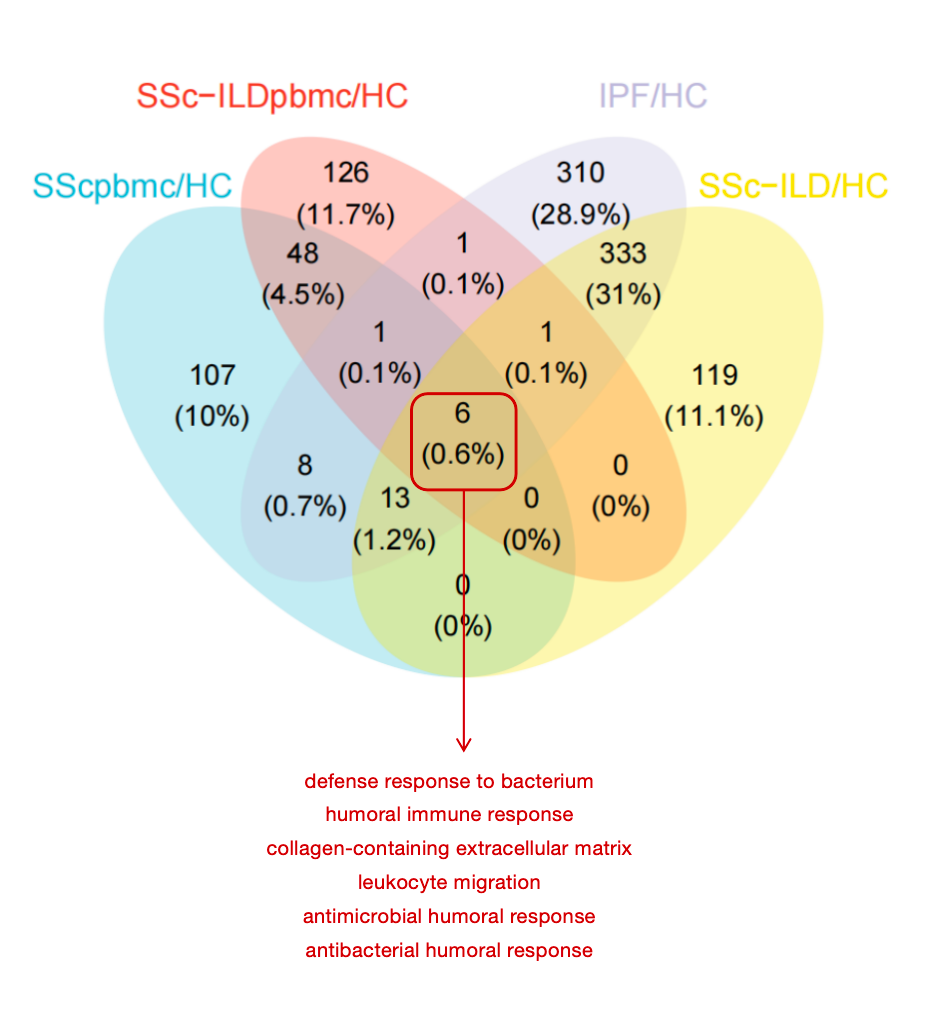

Supplement: Supplementary file 12 — Supplementary Files 12 Venn plot: The intersection of pathways of upregulated genes among the four groups SSc versus control (pbm), SSc‐ILD versus control (pbmc), IPF versus control (lung), and SSc‐ILD versus control (lung) was analyzed. [file CRJ-19-e70072-s012.tif]
