# Supplementary material for: Immunological Features and Potential Biomarkers of Systemic Sclerosis–Associated Interstitial Lung Disease and Idiopathic Pulmonary Fibrosis
Source: Clin Respir J. 2025 Mar 31;19(4):e70072. doi: 10.1111/crj.70072 (PMC11959098; doi:10.1111/crj.70072)
Supplement: Supplementary file 13 — Supplementary Files 13 Basic clinical data of samples used for Q‐PCR. [file CRJ-19-e70072-s011.docx]

|  | IPF(n=6) | Control(n=6) | P value |
| --- | --- | --- | --- |
| Sex (Male,%) | 3 (50%) | 4 (67%) | 0.500 |
| Age | 60.41±11.60 | 58.77±11.94 | 0.113 |
| Smoking | | | 0.455 |
| Never | 0 | 2 |  |
| Ever | 6 | 4 |  |
| Enrollment FVC | 2.35±1.09 | NA | 0.224 |
| Enrollment FVC% | 67.62±18.18 | NA | 0.157 |
| Enrollment DLCO a | 10.97±4.22 | NA | 0.211 |
| Enrollment DLCO% a | 44.33±11.18 | NA | 0.160# |

Additional File 12 Clinical data for validation Q-PCR analysis
